# Supplementary material for: Uracil as a Zn-Binding Bioisostere of the Allergic Benzenesulfonamide in the Design of Quinoline–Uracil Hybrids as Anticancer Carbonic Anhydrase Inhibitors
Source: Pharmaceuticals (Basel). 2022 Apr 19;15(5):494. doi: 10.3390/ph15050494 (PMC9146896; doi:10.3390/ph15050494)
Supplement: Supplementary file 1 [file pharmaceuticals-15-00494-s001.zip › pharmaceuticals-1672236-supplementary.pdf]

Supporting information

Uracil as a Zn-Binding Bioisostere in the Design of Quinoline-Uracil Hybrids as Anticancer Carbonic Anhydrase Inhibitors.

Samar A. El-Kalyoubi[1], Ehab S. Taher[2], Tarek S. Ibrahim[3,4]*, Mohammed Farrag El-Behairy[5], Amany M. M. Al-Mahmoudy[4].

[1]Department of Pharmaceutical Organic Chemistry, Faculty of Pharmacy (Girls), Al-Azhar University, Nasr City, 11651, Cairo, Egypt. SamarEl-kalyoubi.52@azhar.edu.eg
[2] Department of Pharmaceutical Organic Chemistry, Faculty of Pharmacy, Al-Azhar University, Assiut, 71524, Egypt. ehabtaher@azhar.edu.eg
[3]Department of Pharmaceutical Chemistry, Faculty of Pharmacy, King Abdulaziz University, Jeddah, 21589, Saudi Arabia.
[4]Department of Pharmaceutical Organic Chemistry, Faculty of Pharmacy, Zagazig University, Zagazig, 44519, Egypt. amanyma@zu.edu.eg
[5]Department of Organic and Medicinal Chemistry, Faculty of Pharmacy, University of Sadat City, Menoufiya, 32897, Egypt. mohammed.farrag@fop.usc.edu.eg

* Correspondence: tmabrahem@kau.edu.sa (T.S.I.)

Table of Contents:

Legend	Figure or table number	Page number
^{1}H NMR and ^{13}C NMR spectra of (*E*)-6-amino-5-(((2-oxo-1,2-dihydroquinolin-3-yl)methylene)amino)pyrimidine-2,4(1H,3H)-dione (10a)	Figure S1	S3
^{1}H NMR and ^{13}C NMR spectra of (*E*)-6-amino-1-benzyl-5-(((2-oxo-1,2-dihydroquinolin-3-yl)methylene)amino)pyrimidine-2,4(1H,3H)-dione (10b)	Figure S2	S4
^{1}H NMR and ^{13}C NMR spectra of (*E*)-6-amino-1-ethyl-5-(((2-oxo-1,2-dihydroquinolin-3-yl)methylene)amino)pyrimidine-2,4(1H,3H)-dione (10c).	Figure S3	S5
^{1}H NMR and ^{13}C NMR spectra of (*E*)-6-amino-1-methyl-5-(((2-oxo-1,2-dihydroquinolin-3-yl)methylene)amino)pyrimidine-2,4(1H,3H)-dione (10d).	Figure S4	S6
^{1}H NMR and ^{13}C NMR spectra of (*E*)-3-(((6-amino-1-methyl-4-oxo-2-thioxo-1,2,3,4-tetrahydropyrimidin-5-yl)imino)methyl) quinolin-2(1H)-one (10e)	Figure S5	S7
^{1}H NMR and ^{13}C NMR spectra of (*E*)-6-amino-1-ethyl-5-(((8-methyl-2-oxo-1,2-dihydroquinolin-3-yl)methylene)amino) pyrimidine-2,4(1H,3H)-dione (10f)	Figure S6	S8
^{1}H NMR and ^{13}C NMR spectra of (*E*)-3-(((6-amino-1-methyl-4-oxo-2-thioxo-1,2,3,4-tetrahydropyrimidin-5-yl)imino)methyl)-8-methylquinolin-2(1H)-one (10g)	Figure S7	S9
^{1}H NMR and ^{13}C NMR spectra of (*E*)-6-amino-5-(((6-methoxy-2-oxo-1,2-dihydroquinolin-3-yl)methylene)amino)pyrimidine-2,4(1H,3H)-dione (10h)	Figure S8	S10
^{1}H NMR and ^{13}C NMR spectra of (*E*)-6-amino-1-benzyl-5-(((6-methoxy-2-oxo-1,2-dihydroquinolin-3-yl)methylene)amino) pyrimidine-2,4(1H,3H)-dione (10i)	Figure S9	S11
^{1}H NMR and ^{13}C NMR spectra of (*E*)-6-amino-1-ethyl-5-(((6-methoxy-2-oxo-1,2-dihydroquinolin-3-yl)methylene)amino) pyrimidine-2,4(1H,3H)-dione (10j)	Figure S10	S12
^{1}H NMR and ^{13}C NMR spectra of (*E*)-6-amino-5-(((6-methoxy-2-oxo-1,2-dihydro-quinolin-3-yl)methylene)amino)-1-methyl pyrimidine-2,4(1H,3H)-dione (10k)	Figure S11	S13
^{1}H NMR and ^{13}C NMR spectra of (*E*)-3-(((6-amino-1-methyl-4-oxo-2-thioxo-1,2,3,4-tetrahydropyrimidin-5-yl)imino)methyl)-6-methoxyquinolin-2(1H)-one (10l)	Figure S12	S14
2D & 3D interactions of new compounds with the binding site of the human carbonic anhydrases IX (PDB file 5FL4)	Table S1	S15-S19
Mass spectra for compounds 10a-l	Table S2	S19- S22

Figure S1: ^{1}H NMR and ^{13}C NMR spectra of (*E*)-6-amino-5-(((2-oxo-1,2-dihydroquinolin-3-yl)methylene)amino)pyrimidine-2,4(1H,3H)-dione (10a).

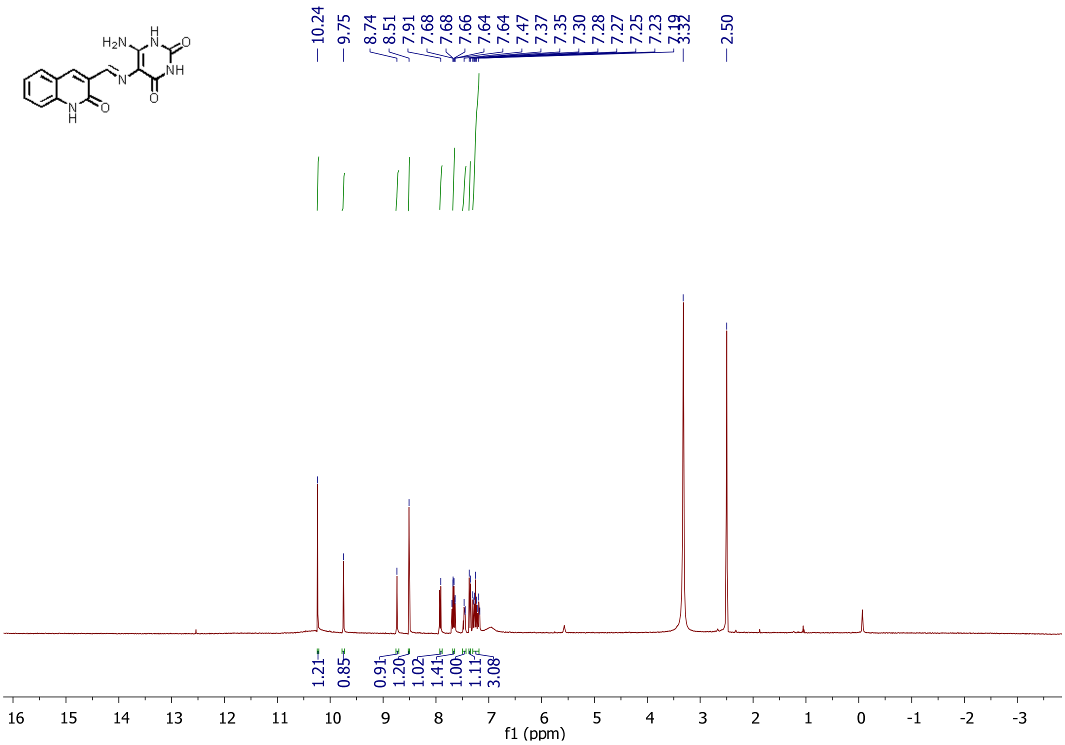

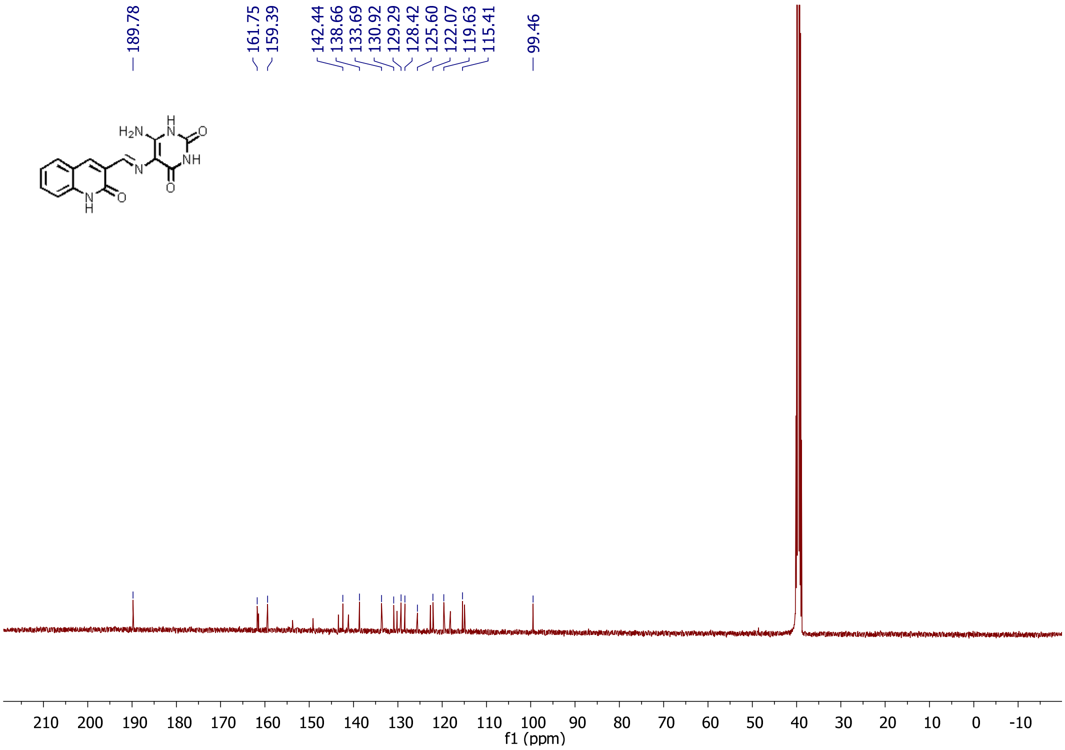

Figure S2: ^{1}H NMR and ^{13}C NMR spectra of (*E*)-6-amino-1-benzyl-5-(((2-oxo-1,2-dihydroquinolin-3-yl)methylene)amino)pyrimidine-2,4(1H,3H)-dione (10b)

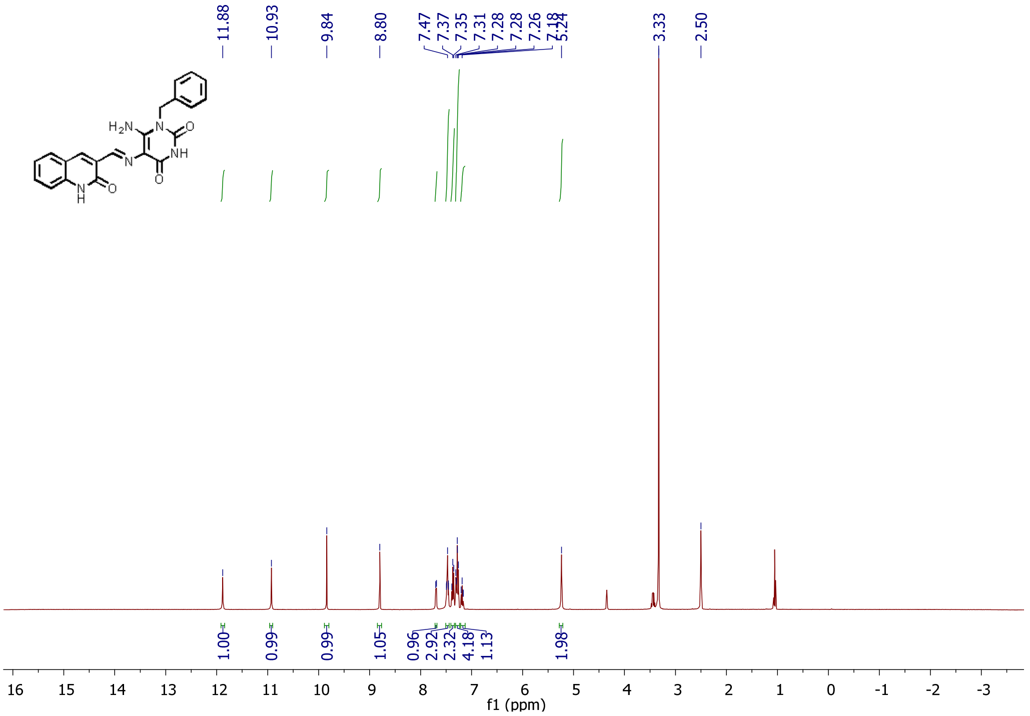

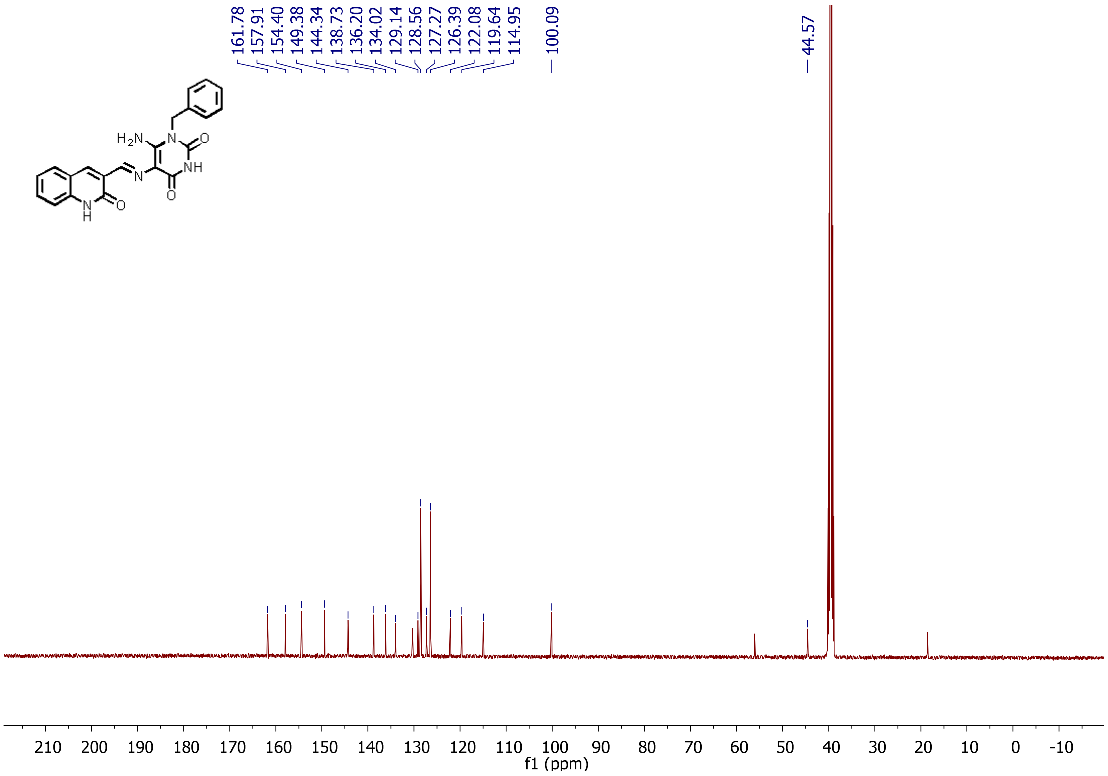

Figure S3: ^{1}H NMR and ^{13}C NMR spectra of (*E*)-6-amino-1-ethyl-5-(((2-oxo-1,2-dihydroquinolin-3-yl)methylene)amino)pyrimidine-2,4(1H,3H)-dione (10c).

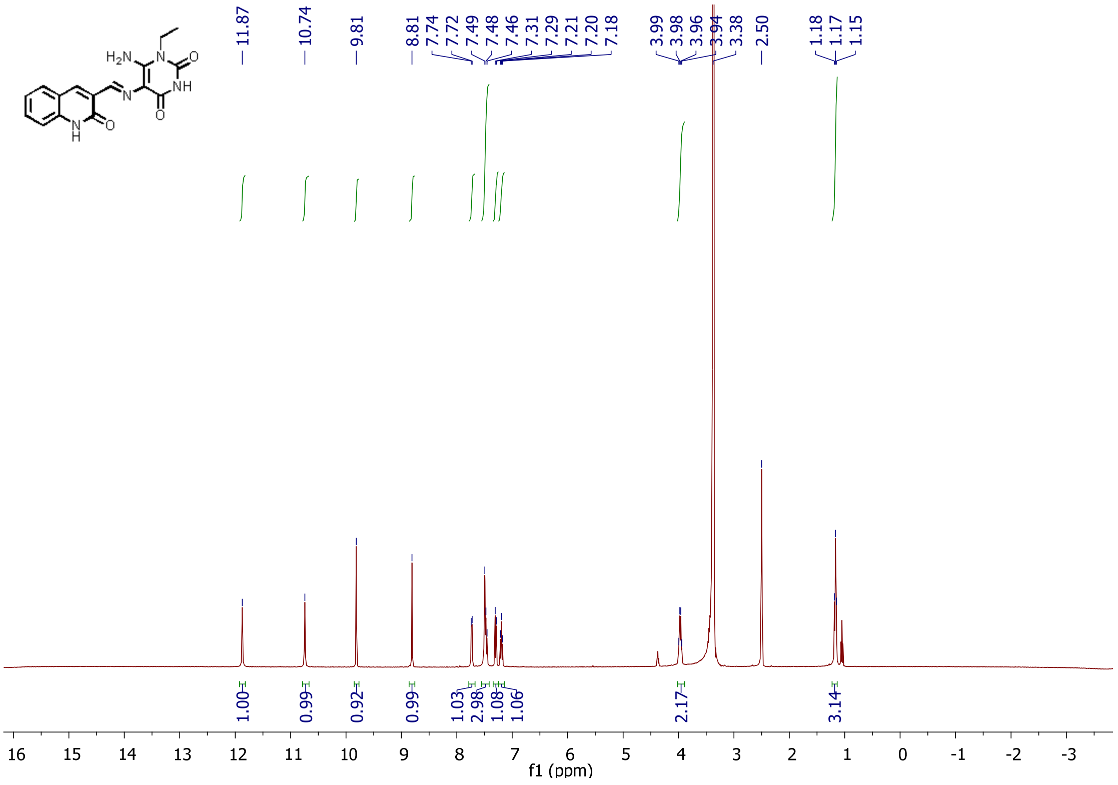

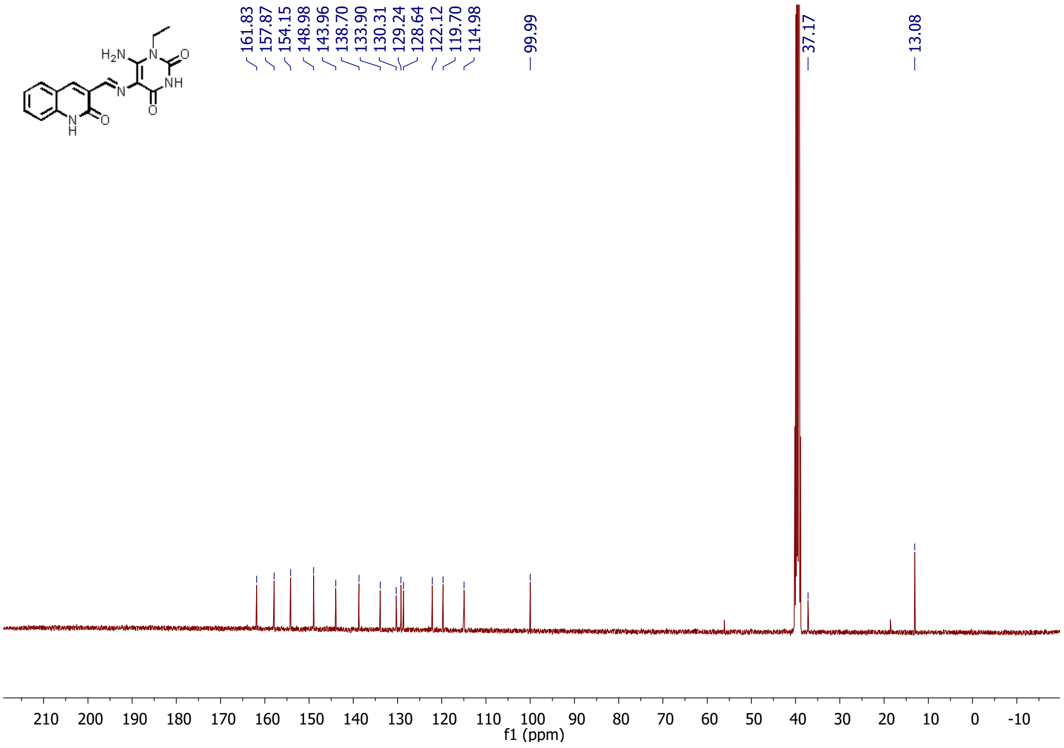

Figure S4: ^{1}H NMR and ^{13}C NMR spectra of (*E*)-6-amino-1-methyl-5-(((2-oxo-1,2-dihydroquinolin-3-yl)methylene)amino)pyrimidine-2,4(1H,3H)-dione (10d).

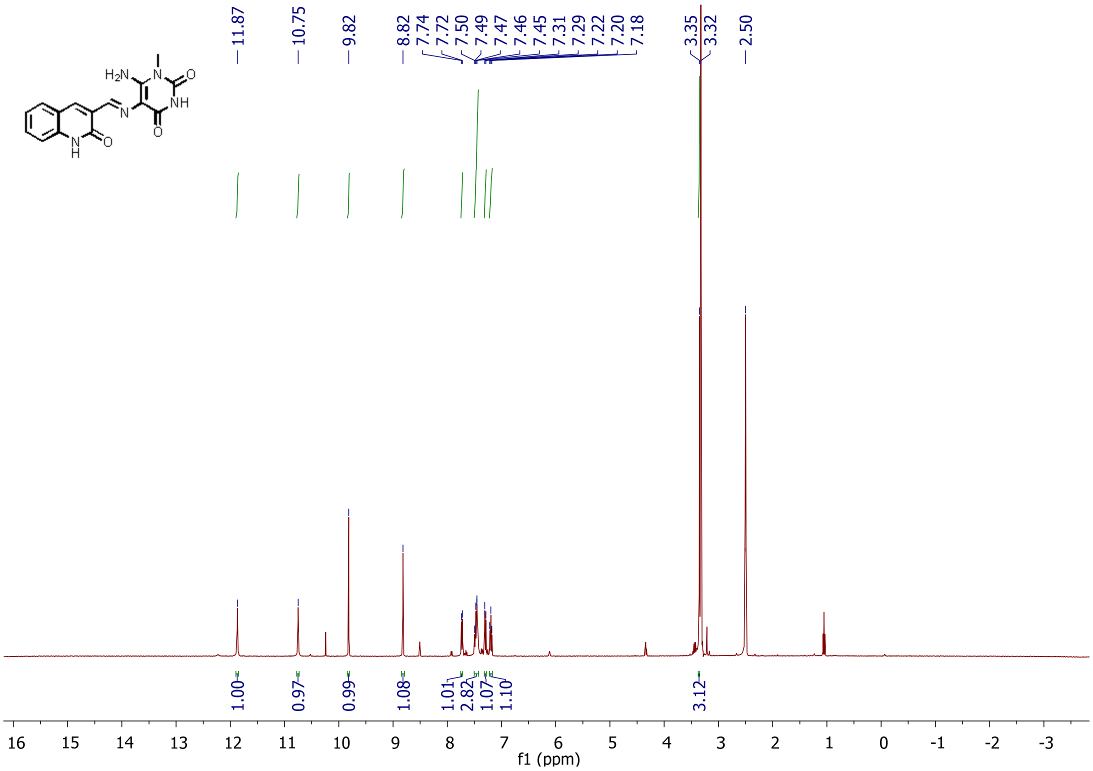

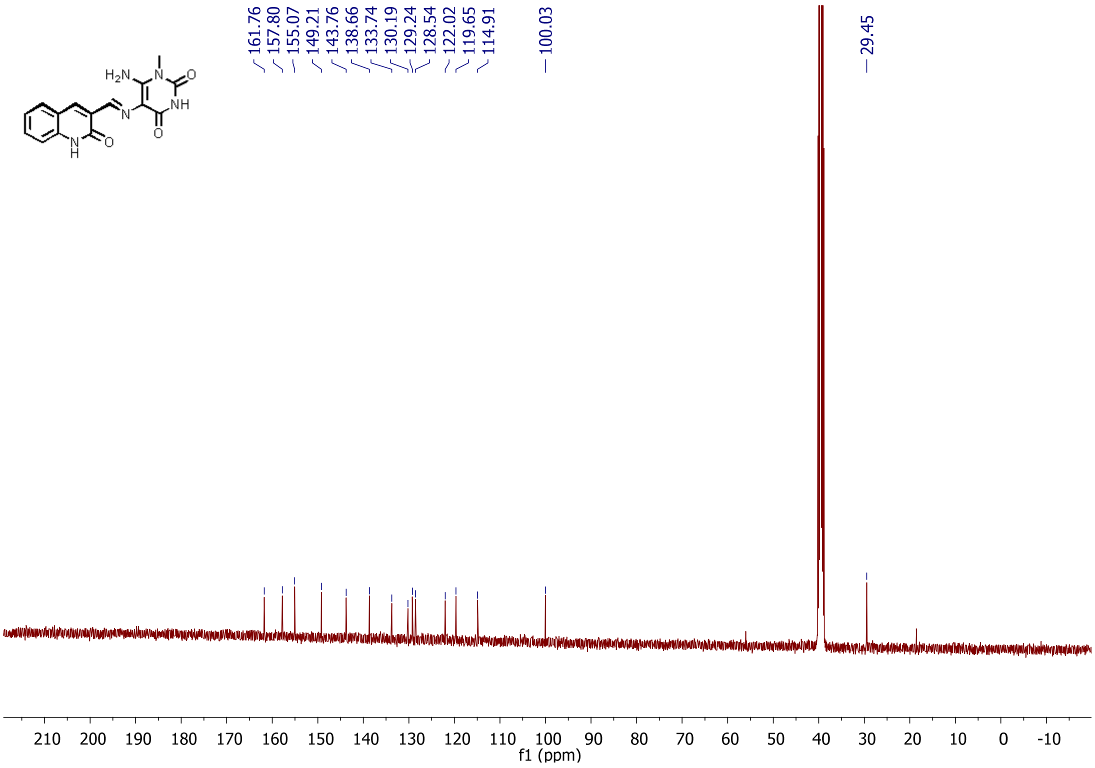

Figure S5: ^{1}H NMR and ^{13}C NMR spectra of (*E*)-3-(((6-amino-1-methyl-4-oxo-2-thioxo-1,2,3,4-tetrahydropyrimidin-5-yl)imino)methyl) quinolin-2(1H)-one (10e)

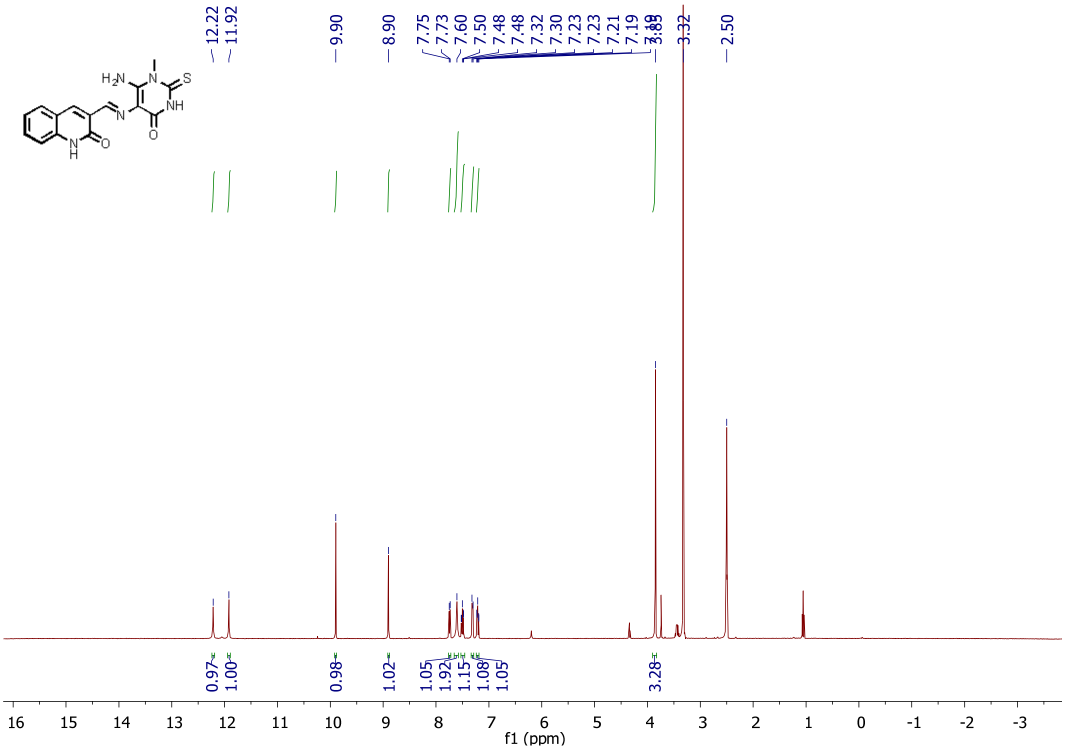

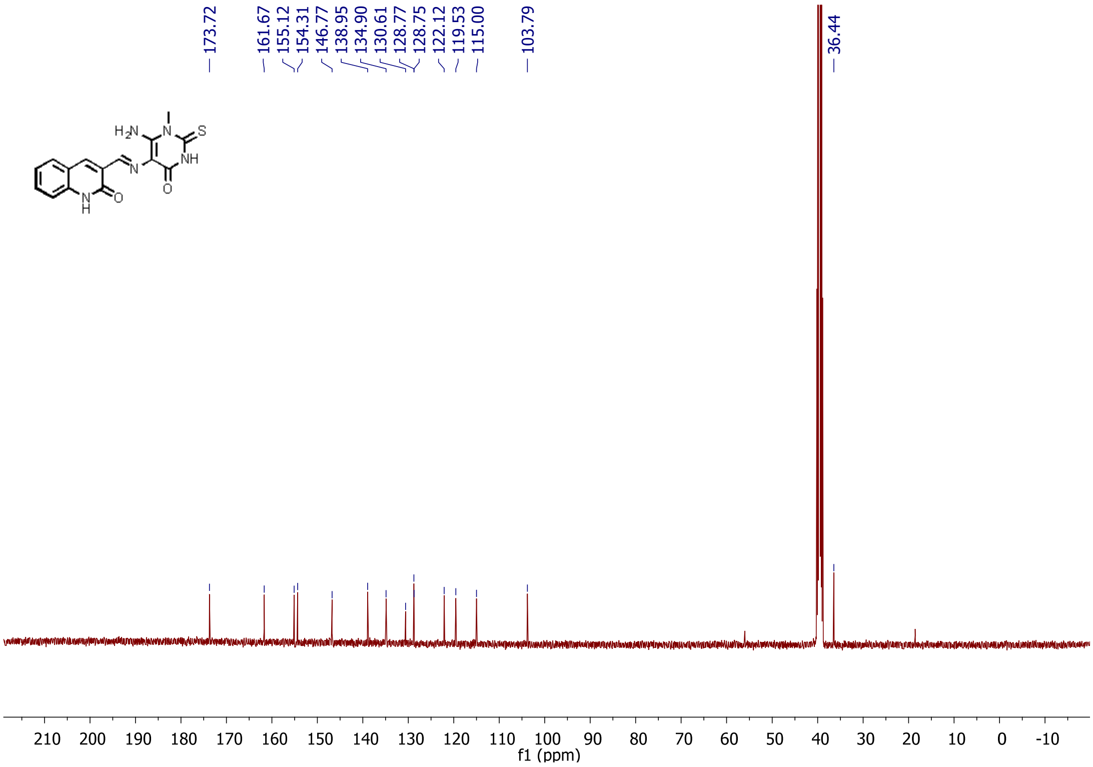

Figure S6: ^{1}H NMR and ^{13}C NMR spectra of (*E*)-6-amino-1-ethyl-5-(((8-methyl-2-oxo-1,2-dihydroquinolin-3-yl)methylene)amino) pyrimidine-2,4(1H,3H)-dione (10f)

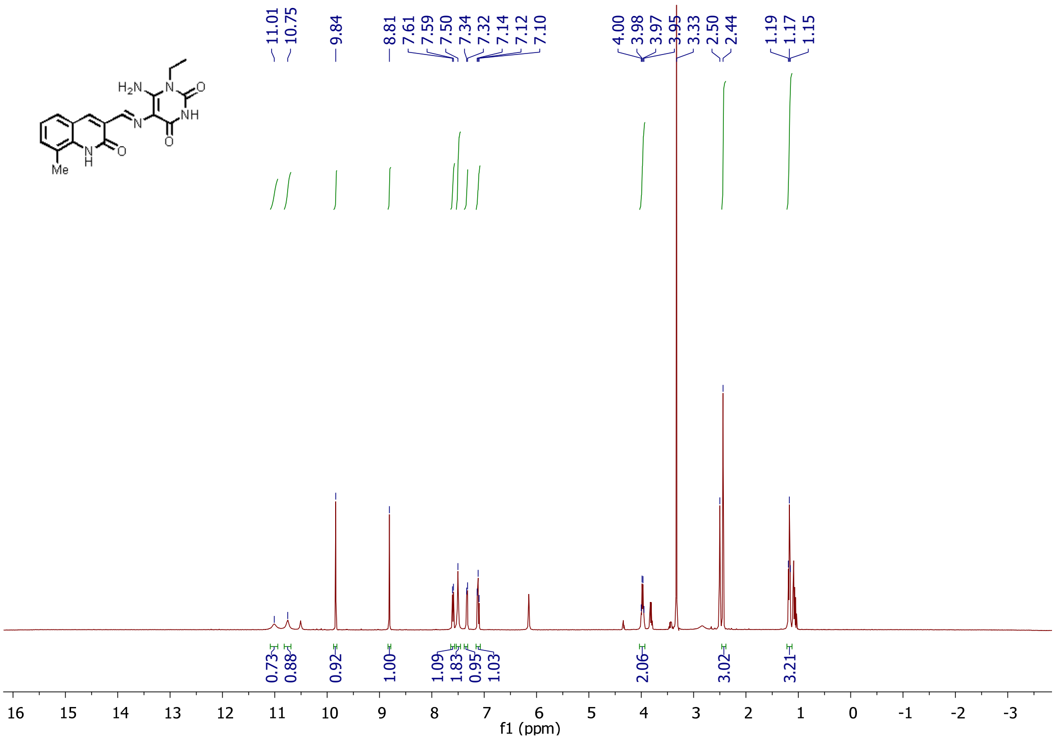

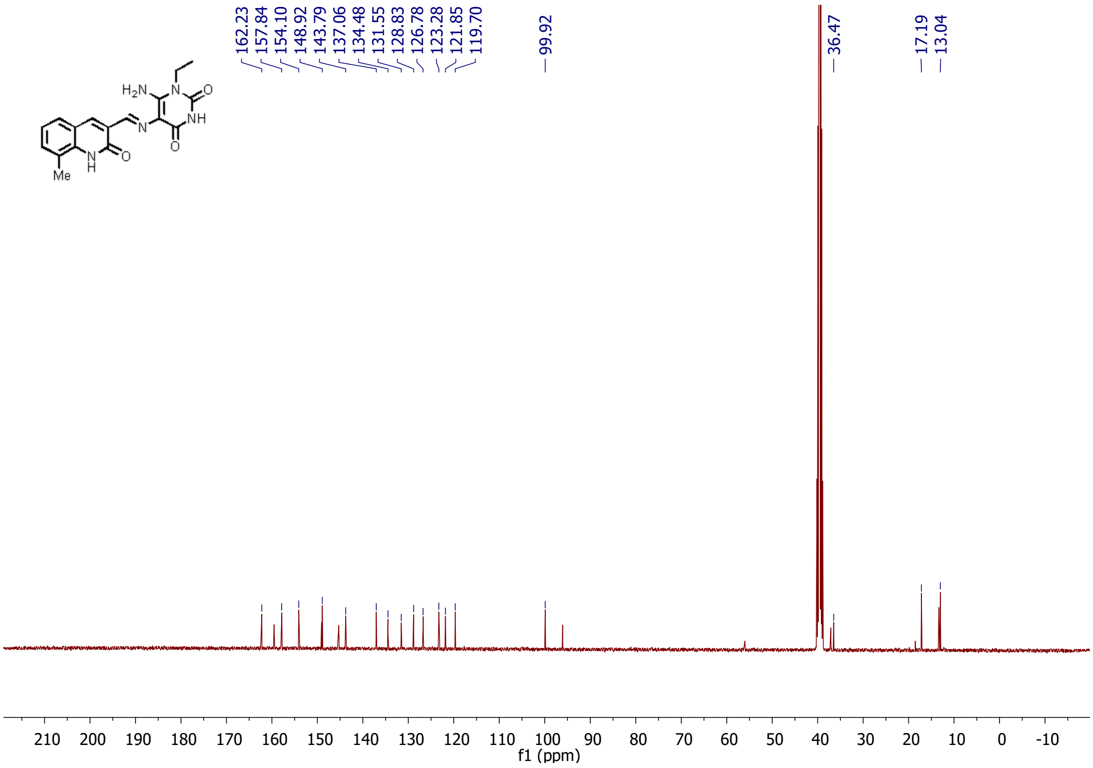

Figure S7: ^{1}H NMR and ^{13}C NMR spectra of (*E*)-3-(((6-amino-1-methyl-4-oxo-2-thioxo-1,2,3,4-tetrahydropyrimidin-5-yl)imino)methyl)-8-methylquinolin-2(1H)-one (10g)

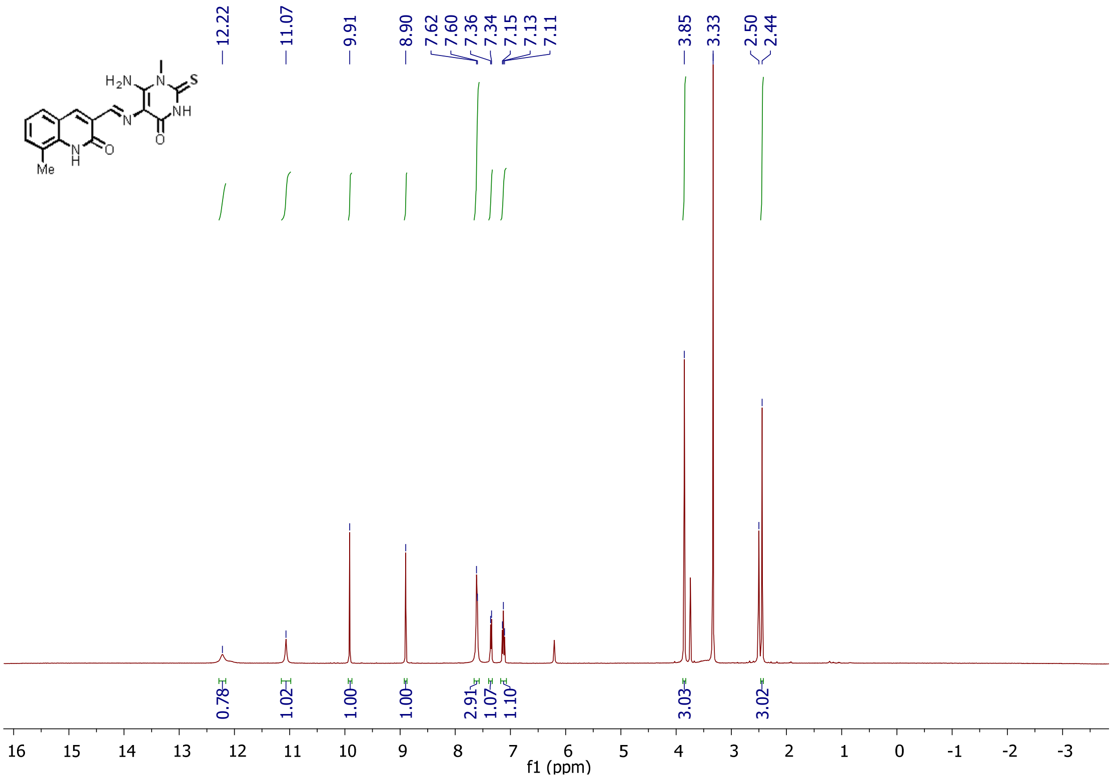

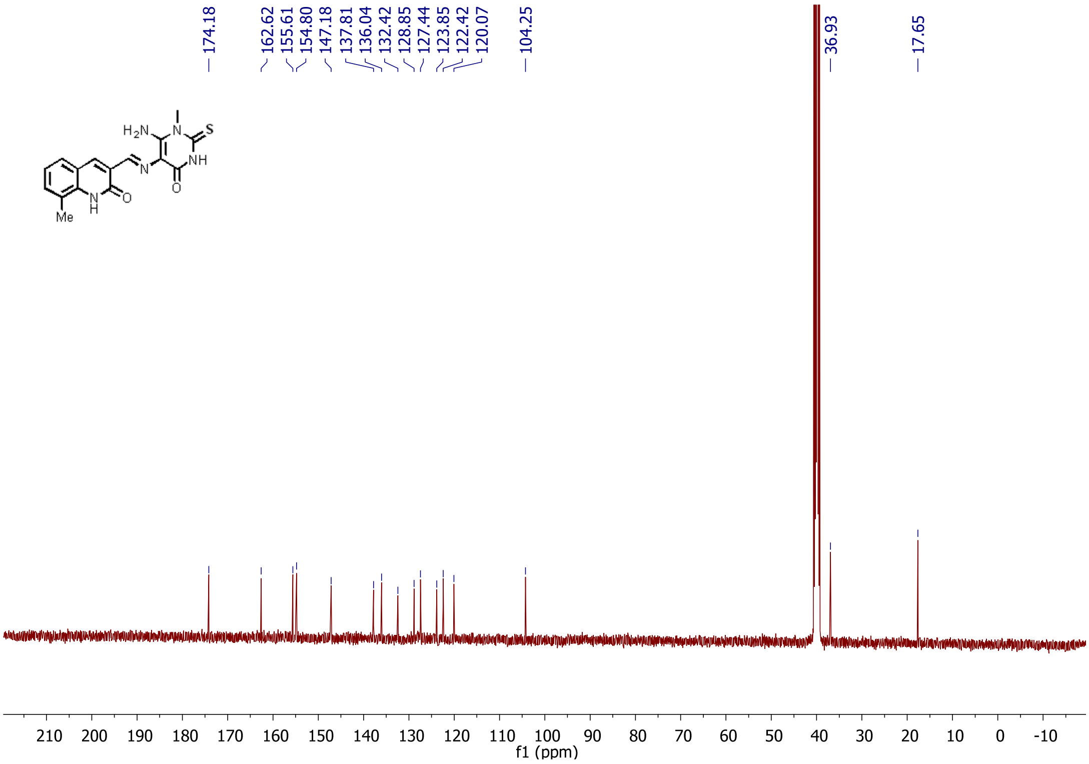

Figure S8: ^{1}H NMR and ^{13}C NMR spectra of (*E*)-6-amino-5-(((6-methoxy-2-oxo-1,2-dihydroquinolin-3-yl)methylene)amino)pyrimidine-2,4(1H,3H)-dione (10h)

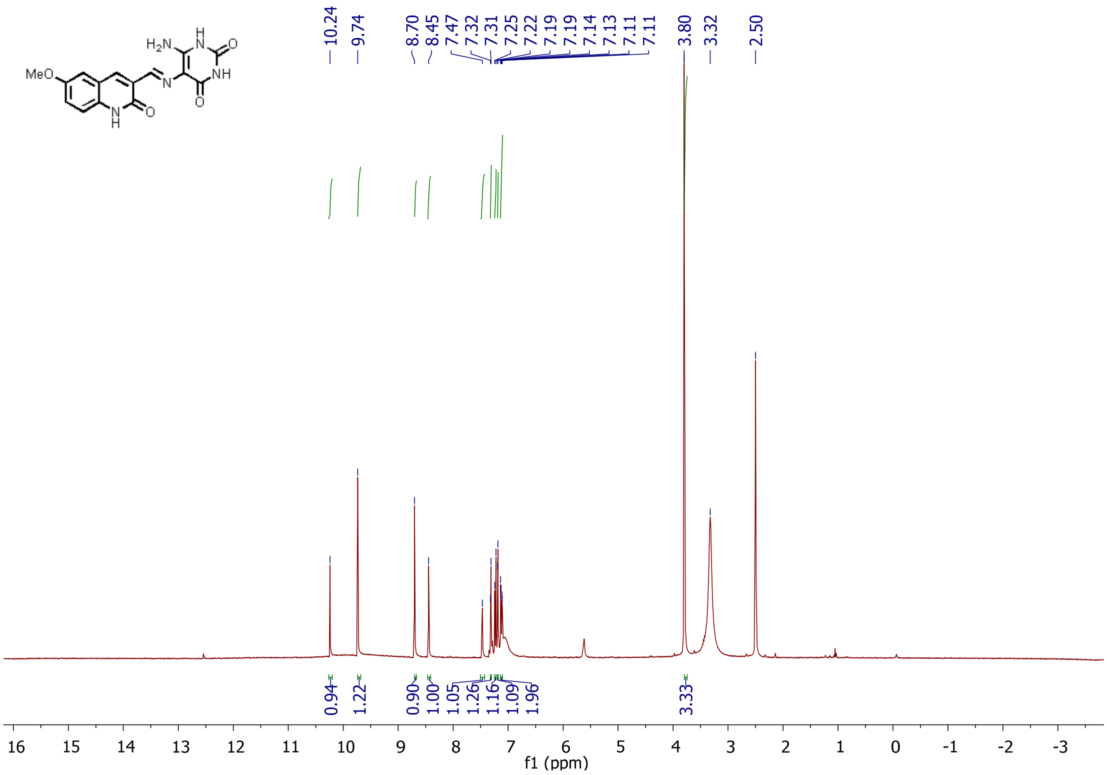

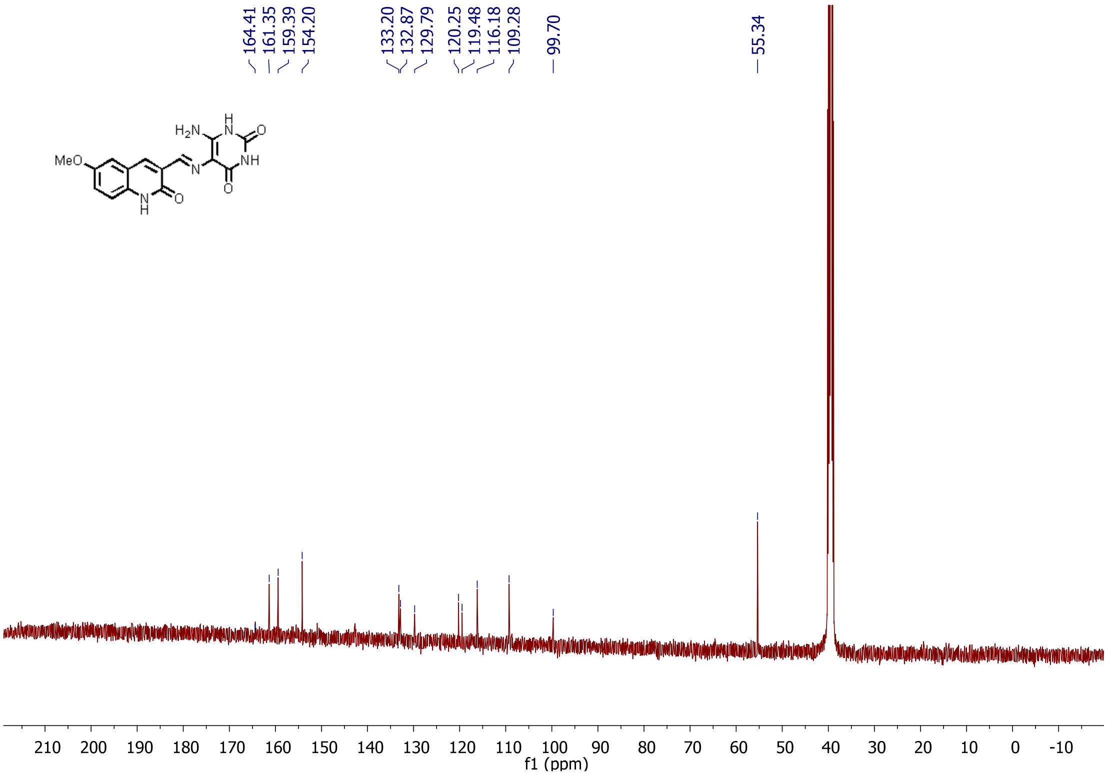

Figure S9: ^{1}H NMR and ^{13}C NMR spectra of (*E*)-6-amino-1-benzyl-5-(((6-methoxy-2-oxo-1,2-dihydroquinolin-3-yl)methylene)amino) pyrimidine-2,4(1H,3H)-dione (10i)

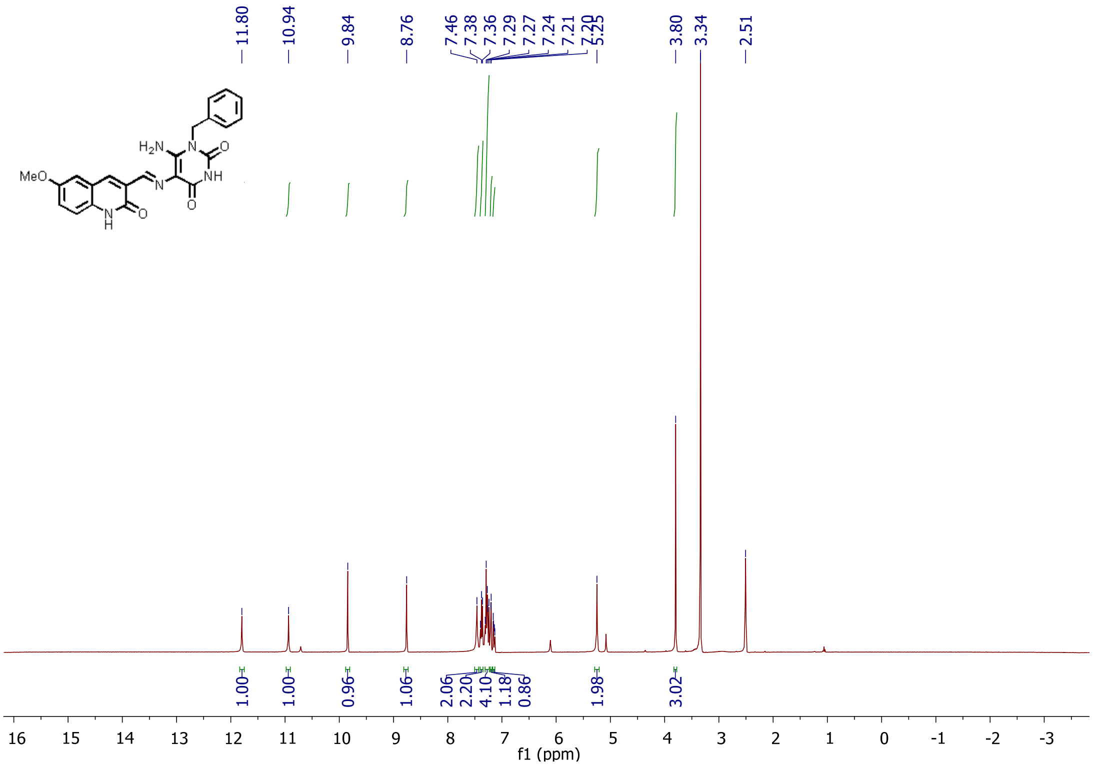

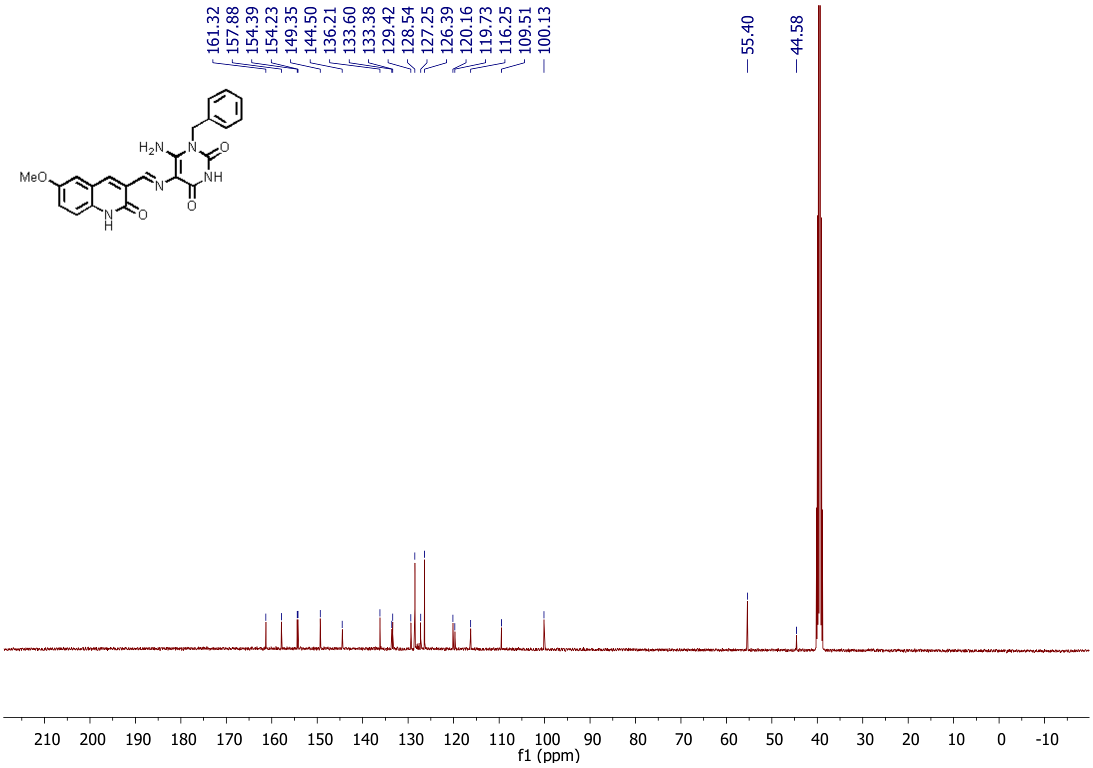

Figure S10: ^{1}H NMR and ^{13}C NMR spectra of (*E*)-6-amino-1-ethyl-5-(((6-methoxy-2-oxo-1,2-dihydroquinolin-3-yl)methylene)amino) pyrimidine-2,4(1H,3H)-dione (10j)

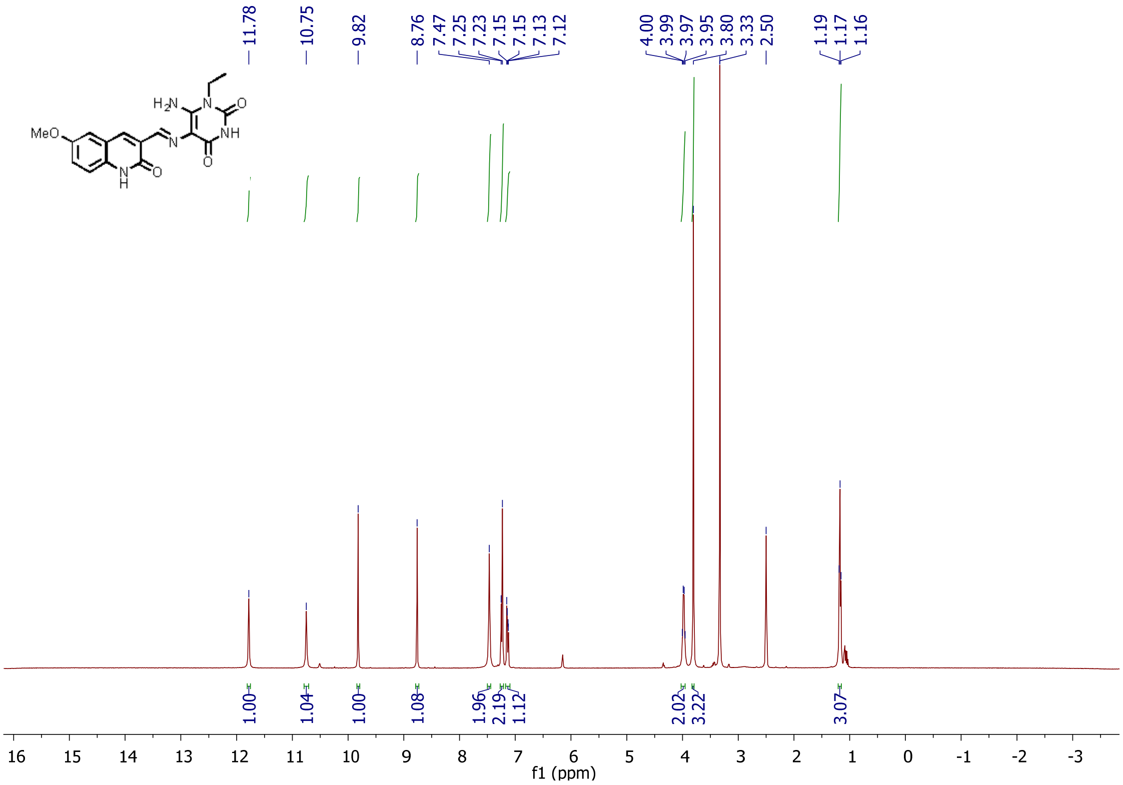

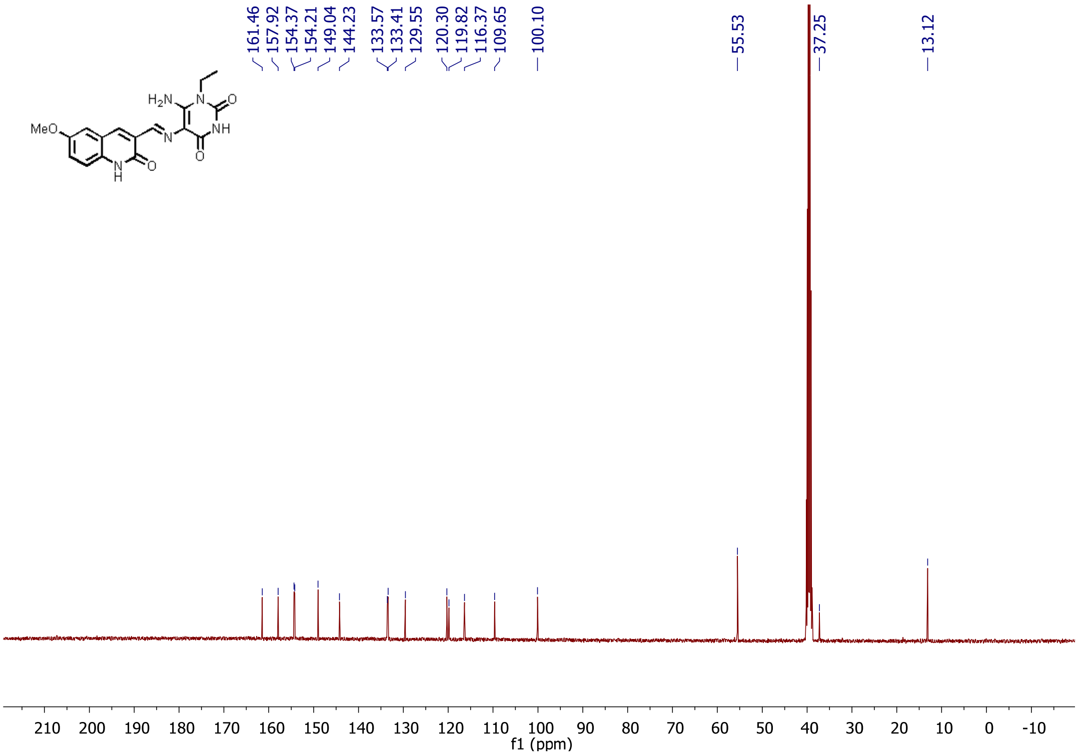

Figure S11: ^{1}H NMR and ^{13}C NMR spectra of (*E*)-6-amino-5-(((6-methoxy-2-oxo-1,2-dihydro-quinolin-3-yl)methylene)amino)-1-methyl pyrimidine-2,4(1H,3H)-dione (10k)

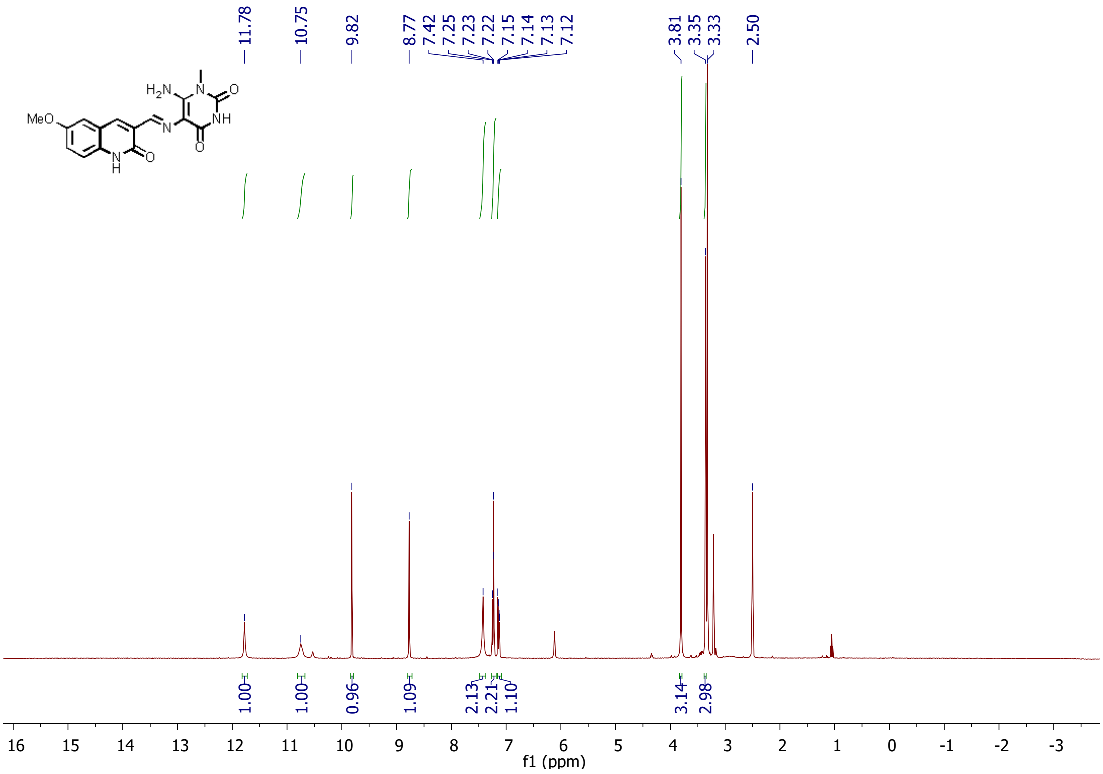

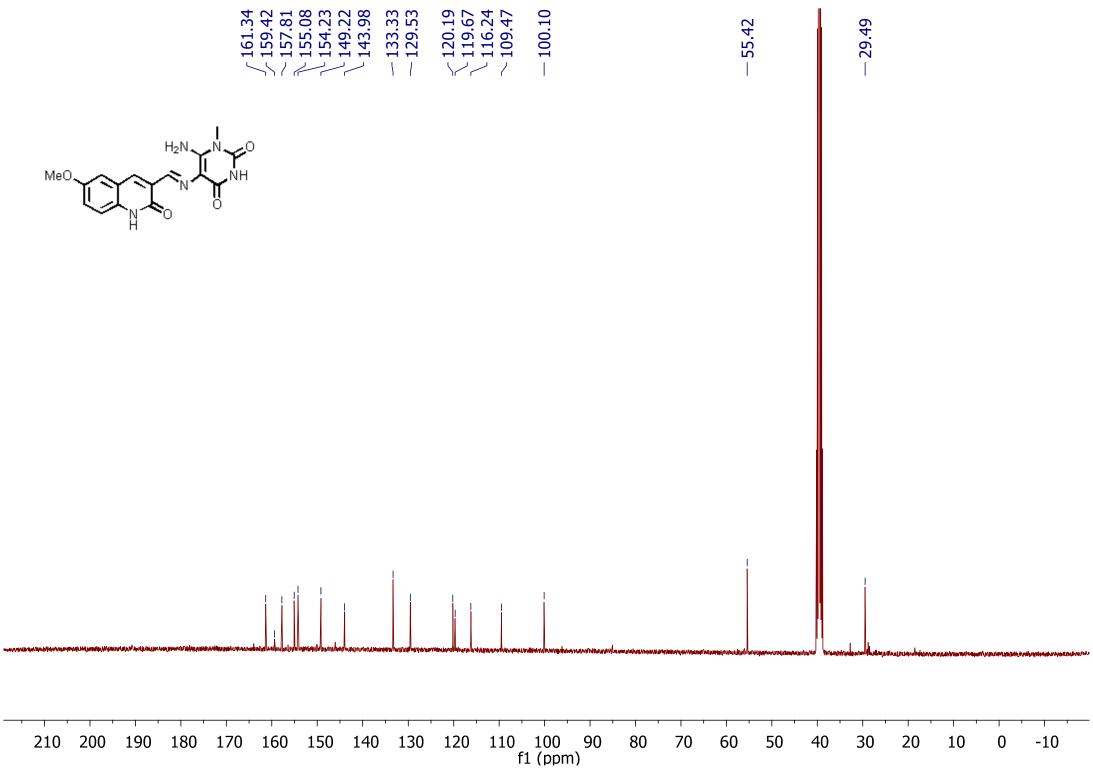

Figure S12: ^{1}H NMR and ^{13}C NMR spectra of (*E*)-3-(((6-amino-1-methyl-4-oxo-2-thioxo-1,2,3,4-tetrahydropyrimidin-5-yl)imino)methyl)-6-methoxyquinolin-2(1H)-one (10l)

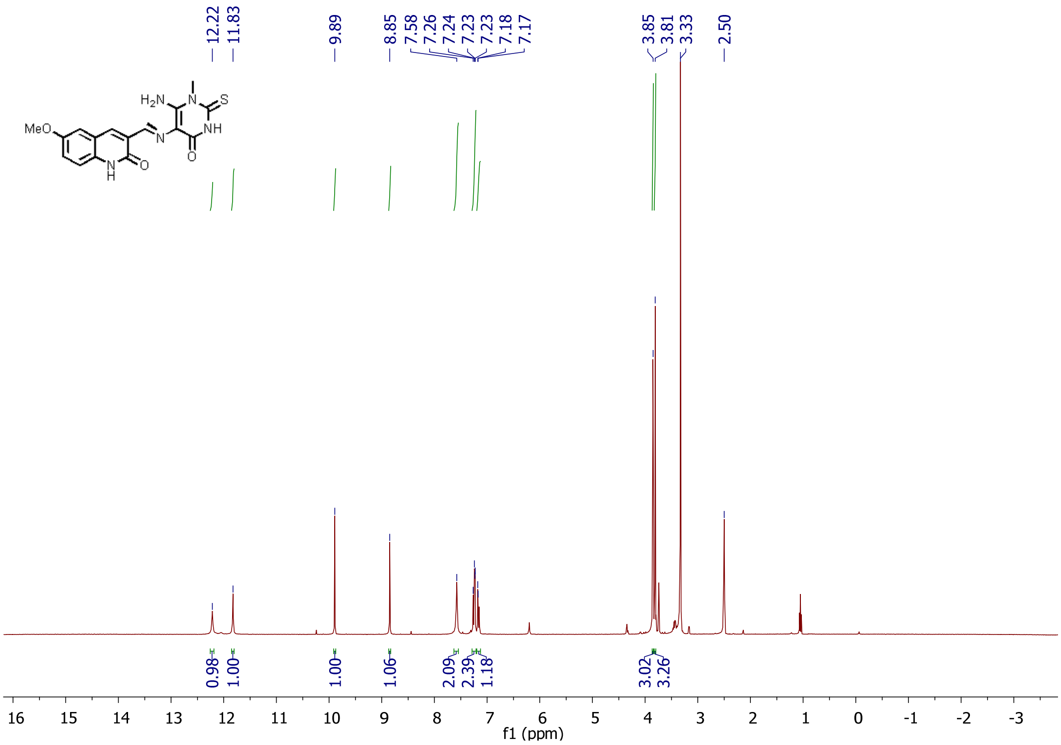

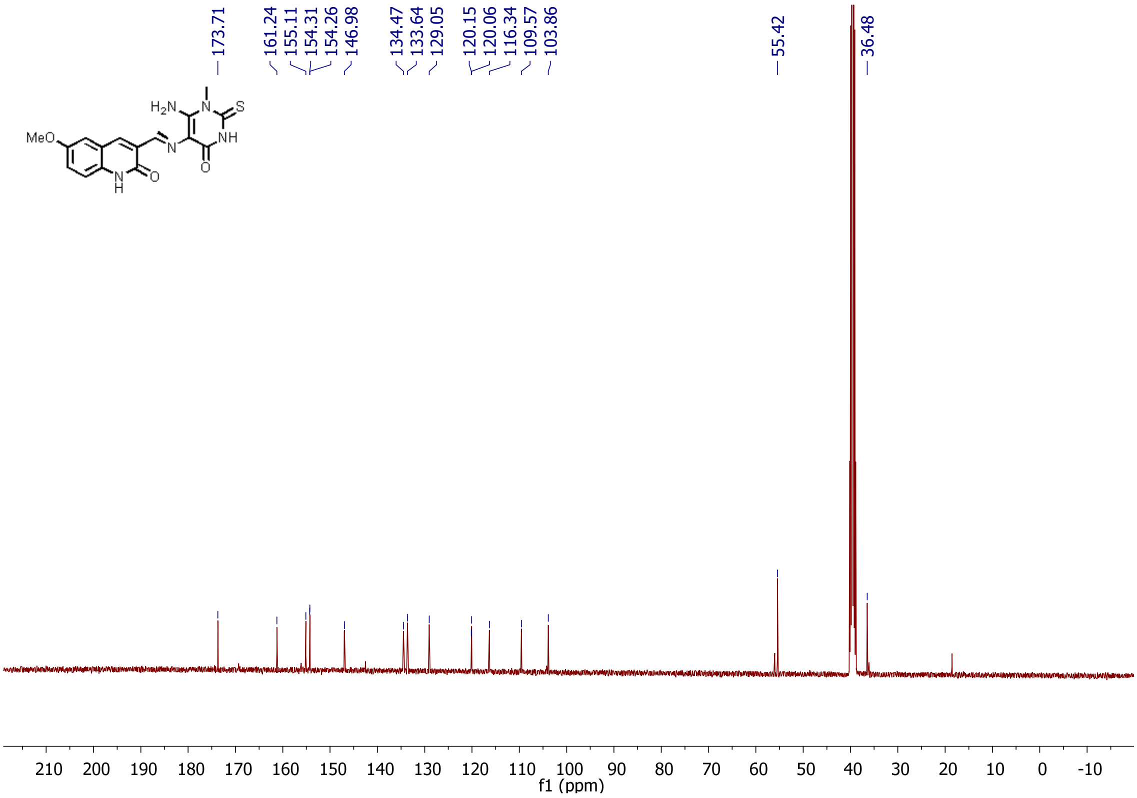

Table S1. 2D & 3D interactions of new compounds with the binding site of the human carbonic anhydrases IX (PDB file 5FL4)

Compound	2D Interaction	3D Interaction
**10a**	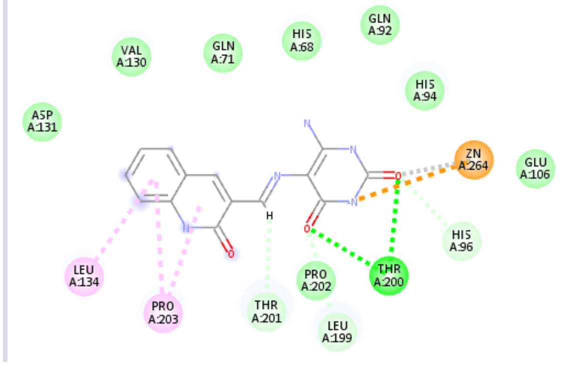	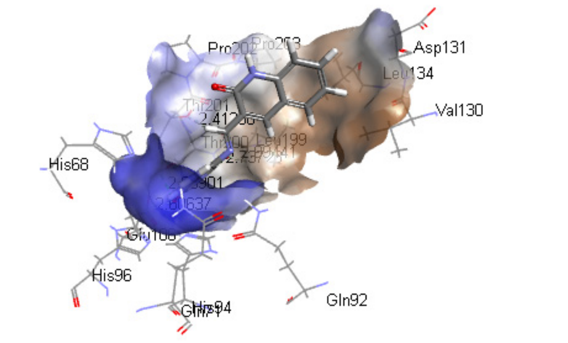
**10b**	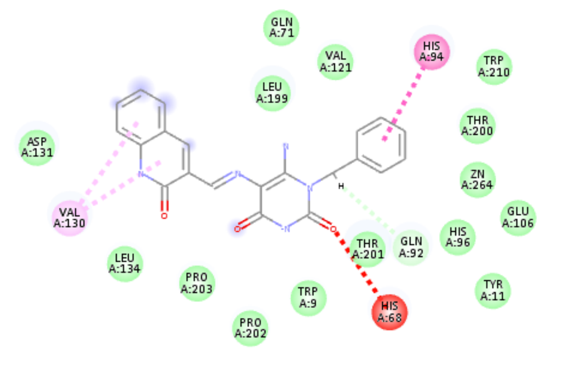	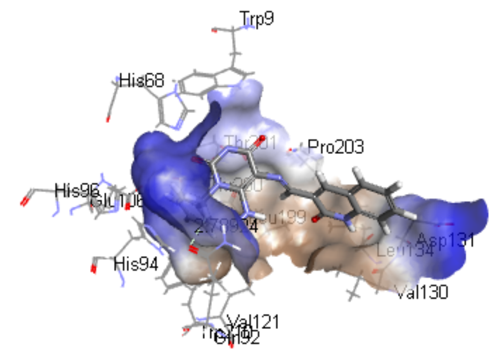
**10c**	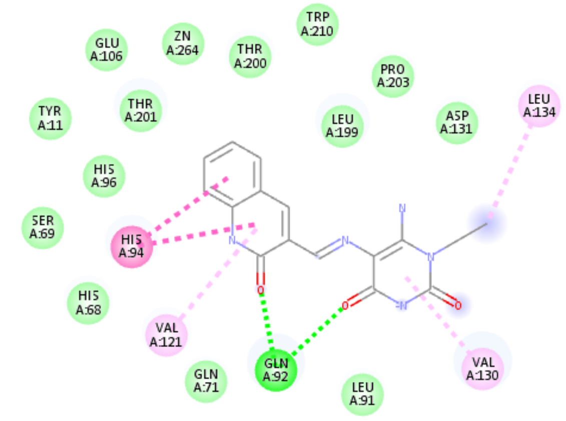	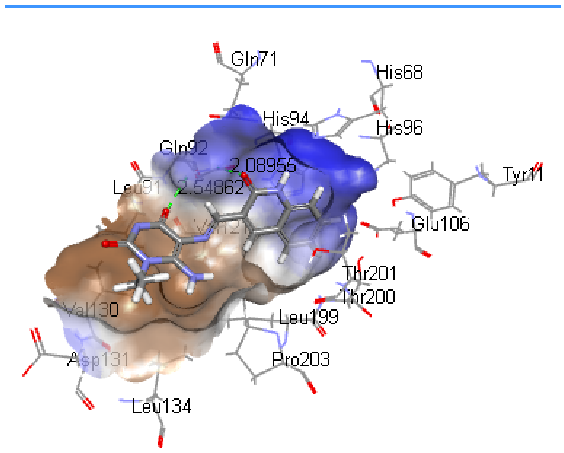

10d

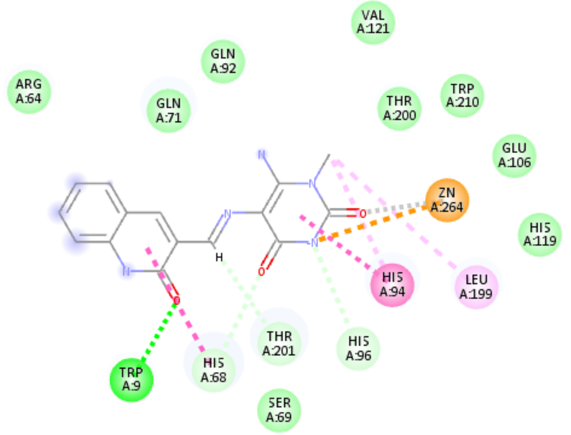
VAL A:121
GLN A:92
ARG A:64
GLN A:71
THR A:200
TRP A:210
GLU A:106
ZN A:264
HIS A:119
HIS A:94
LEU A:199
THR A:201
HIS A:96
TRP A:9
HIS A:68
SER A:69

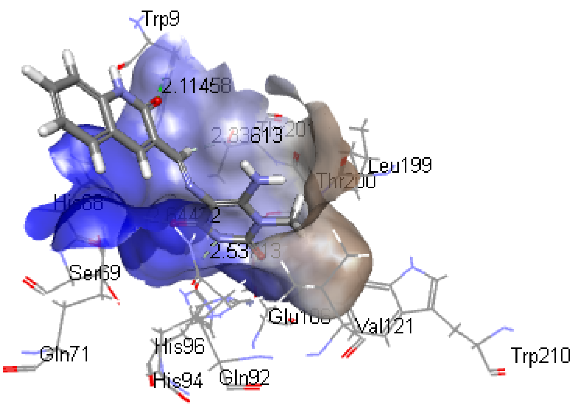
Trp9
2.11458
Leu199
Thr200
Ser69
Val121
Gln71
His96
His94
Gln92
Trp210

10e

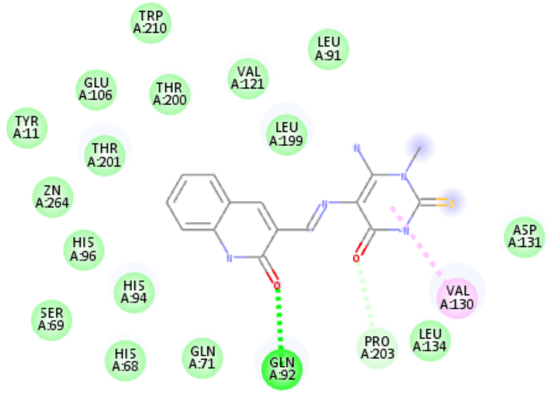
TRP A:210
LEU A:91
VAL A:121
GLU A:106
THR A:200
TYR A:11
THR A:201
LEU A:199
ZN A:264
ASP A:131
HIS A:96
HIS A:94
VAL A:130
SER A:69
PRO A:203
LEU A:134
HIS A:68
GLN A:71
GLN A:92

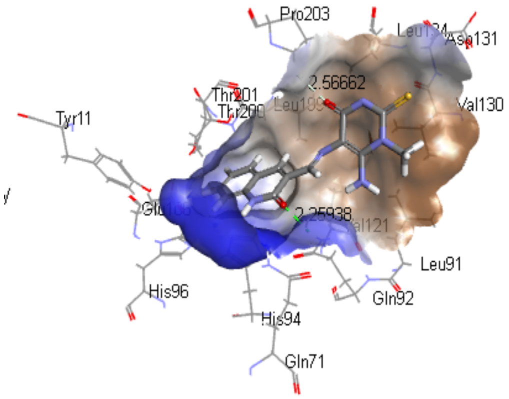
Pro203
2.56662
Tyr11
Thr201
Val130
2.25938
Leu91
His96
Gln92
His94
Gln71

10f

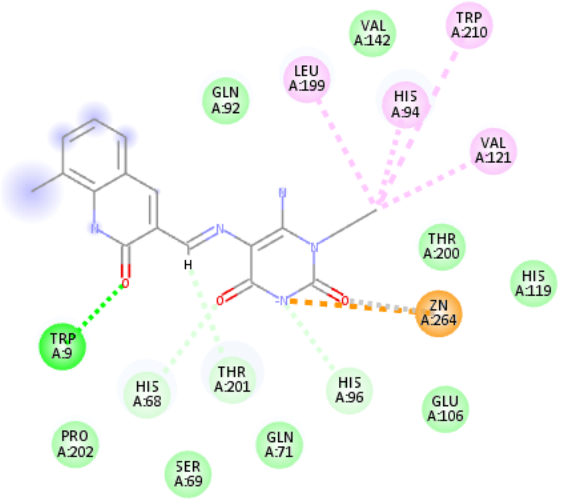
VAL A:142
TRP A:210
LEU A:199
GLN A:92
HIS A:94
VAL A:121
THR A:200
HIS A:119
ZN A:264
TRP A:9
HIS A:68
THR A:201
HIS A:96
GLU A:106
PRO A:202
GLN A:71
SER A:69

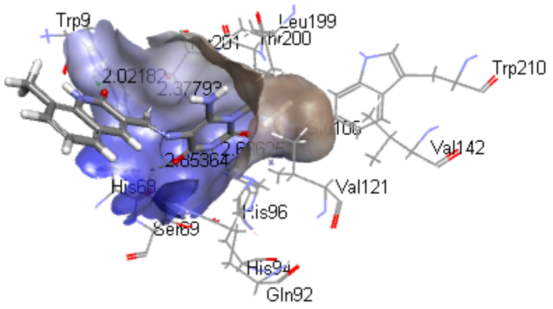
Trp9
Leu199
Trp210
2.02182
Val142
2.65364
Val121
His68
Gln92

10g

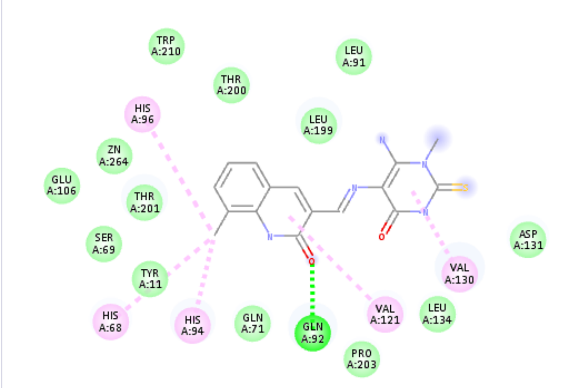
TRP A:210
LEU A:91
THR A:200
HIS A:96
LEU A:199
ZN A:264
GLU A:106
THR A:201
SER A:69
ASP A:131
TYR A:11
VAL A:130
HIS A:68
HIS A:94
GLN A:71
GLN A:92
VAL A:121
LEU A:134
PRO A:203

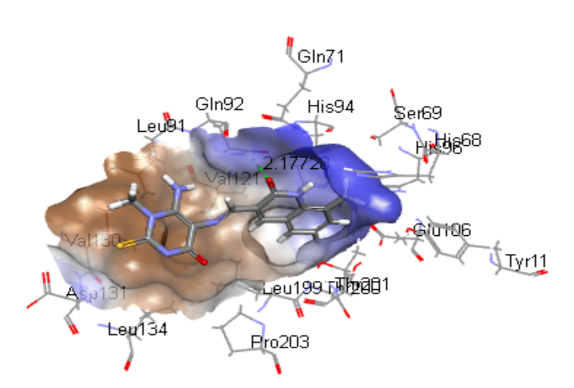
Gln71
Gln92
His94
Ser69
Leu91
Val121
2.17728
Glu106
Tyr11
Val130
Asp131
Leu199
Leu134
Pro203

10h

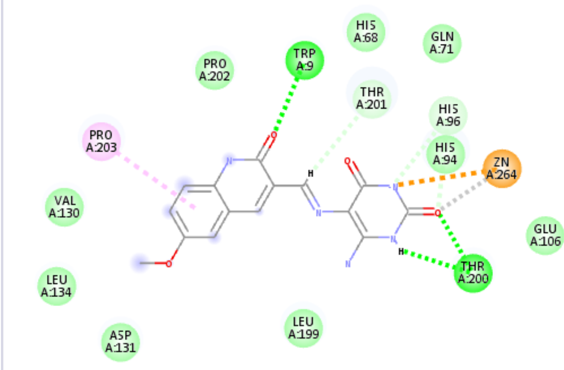
HIS A:68
GLN A:71
PRO A:202
TRP A:9
THR A:201
HIS A:96
PRO A:203
HIS A:94
ZN A:264
VAL A:130
GLU A:106
THR A:200
LEU A:134
LEU A:199
ASP A:131

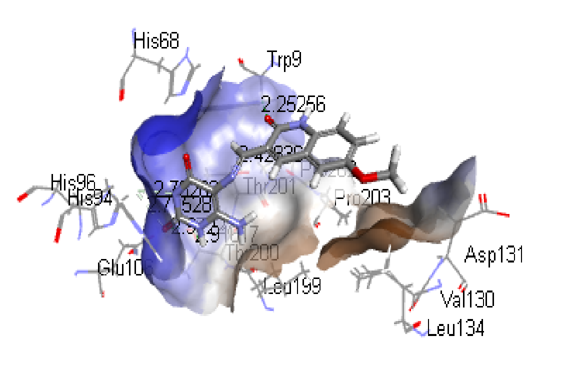
His68
Trp9
2.25256
Thr201
Pro203
His96
His94
Thr200
Asp131
Leu199
Val130
Leu134

10i

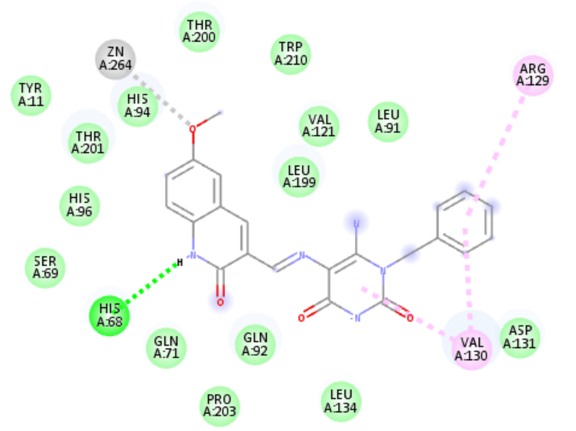
THR A:200
ZN A:264
TRP A:210
ARG A:129
TYR A:11
HIS A:94
VAL A:121
LEU A:91
THR A:201
LEU A:199
HIS A:96
SER A:69
HIS A:68
GLN A:71
GLN A:92
VAL A:130
ASP A:131
PRO A:203
LEU A:134

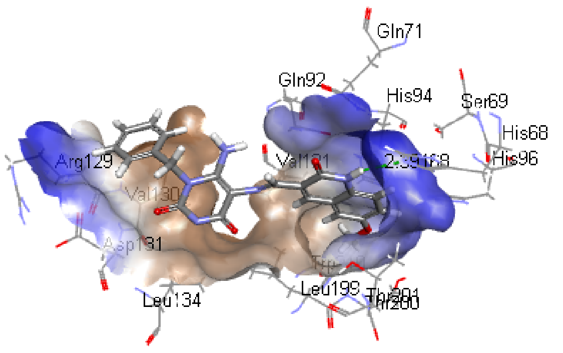
Gln71
Gln92
His94
Ser69
His68
Arg129
His96
Val130
Leu134
Leu199

10j

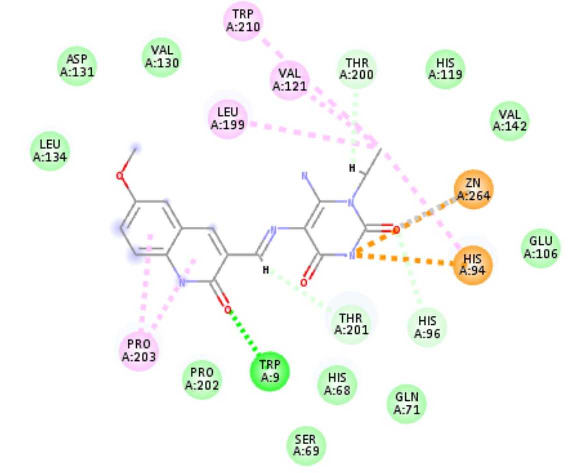

TRP A:210
ASP A:131
VAL A:130
VAL A:121
THR A:200
HIS A:119
LEU A:199
VAL A:142
LEU A:134
ZN A:264
GLU A:106
HIS A:94
THR A:201
HIS A:96
PRO A:203
PRO A:202
TRP A:9
HIS A:68
GLN A:71
SER A:69

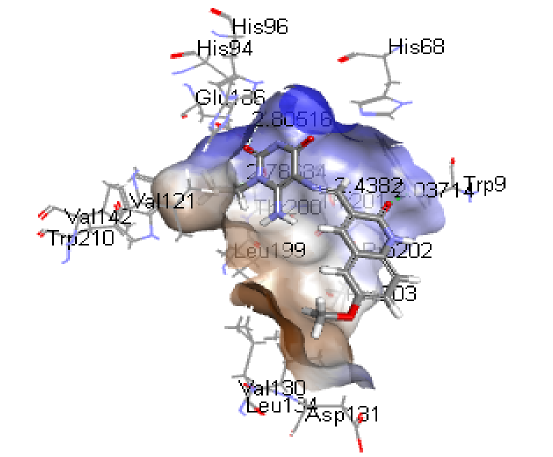

His96
His94
His68
Val142
Val121
Trp210
Leu199
Trp9
Val130
Asp131

10k

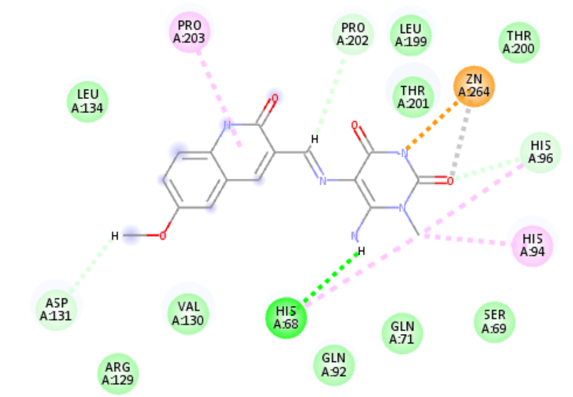

PRO A:203
PRO A:202
LEU A:199
THR A:200
LEU A:134
THR A:201
ZN A:264
HIS A:96
HIS A:94
ASP A:131
VAL A:130
HIS A:68
GLN A:71
SER A:69
ARG A:129
GLN A:92

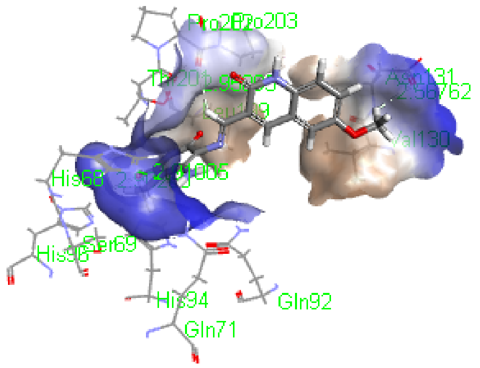

His68
Ser69
His94
Gln92
Gln71

10l

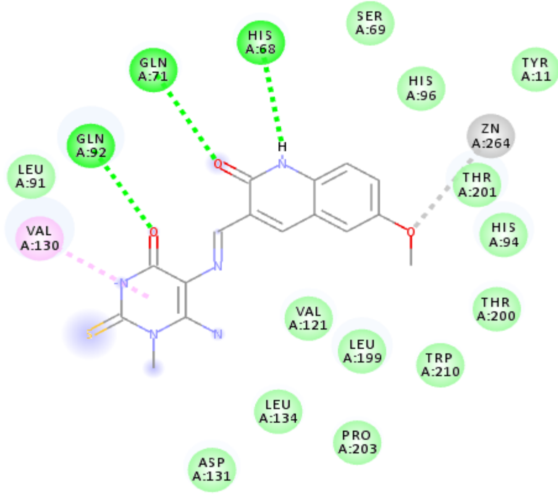

SER A:69
HIS A:68
GLN A:71
TYR A:11
HIS A:96
ZN A:264
GLN A:92
LEU A:91
THR A:201
HIS A:94
VAL A:130
THR A:200
VAL A:121
LEU A:199
TRP A:210
LEU A:134
PRO A:203
ASP A:131

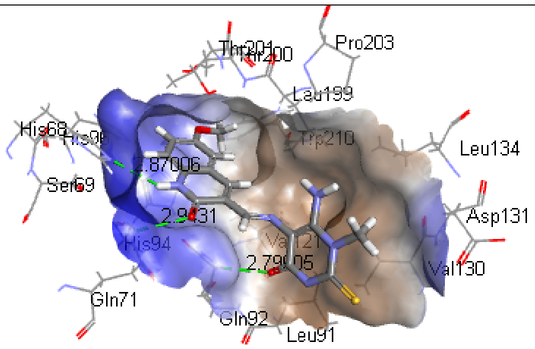

Pro203
Leu199
Leu134
Ser69
Asp131
His94
Val130
Gln71
Gln92
Leu91

**Ligand**	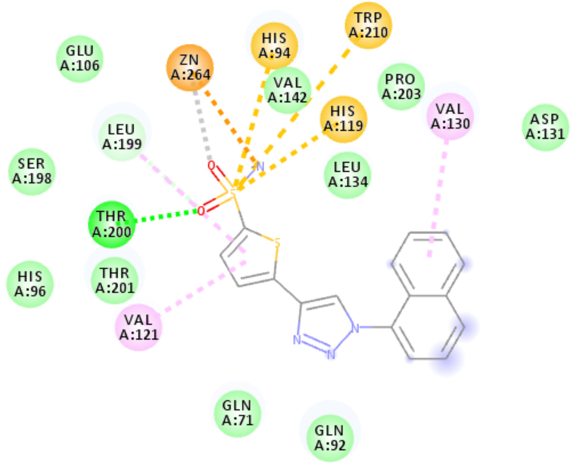	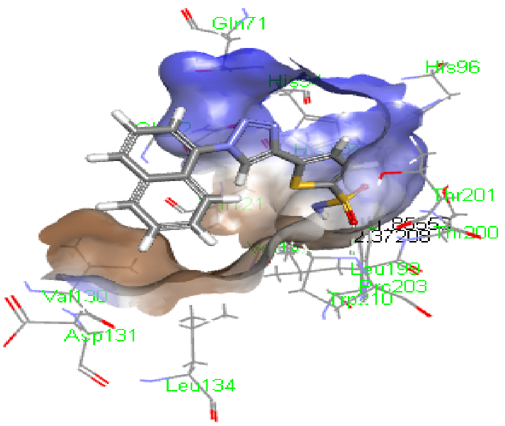

Table S2: Mass spectra for compounds 10a-l

Compound	Mass spectrum
**10a**	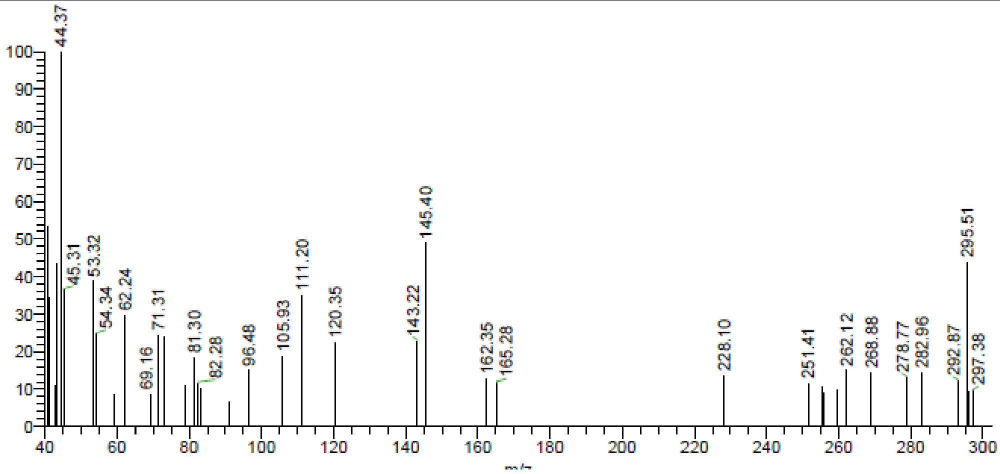

**10b**	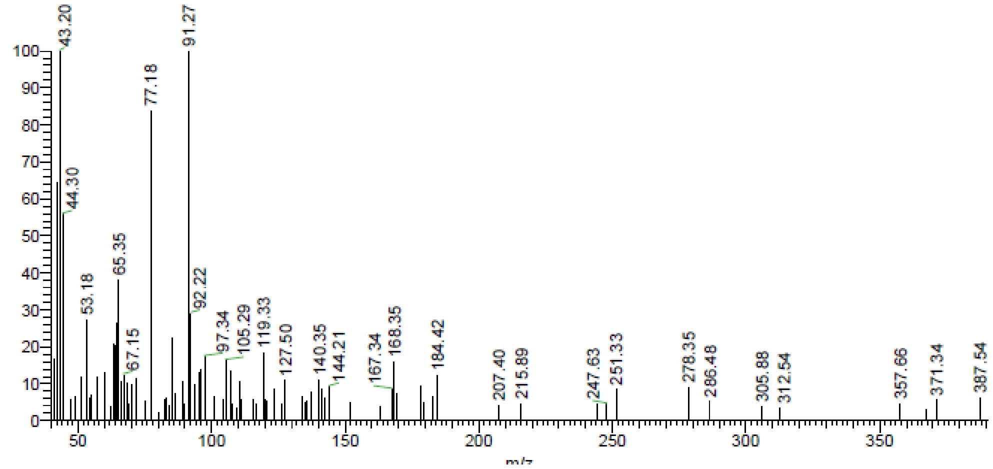
**10c**	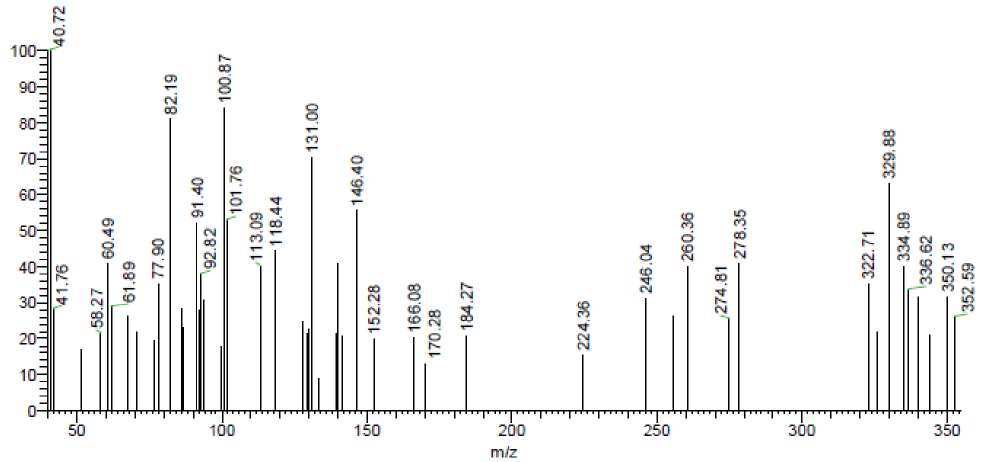
**10d**	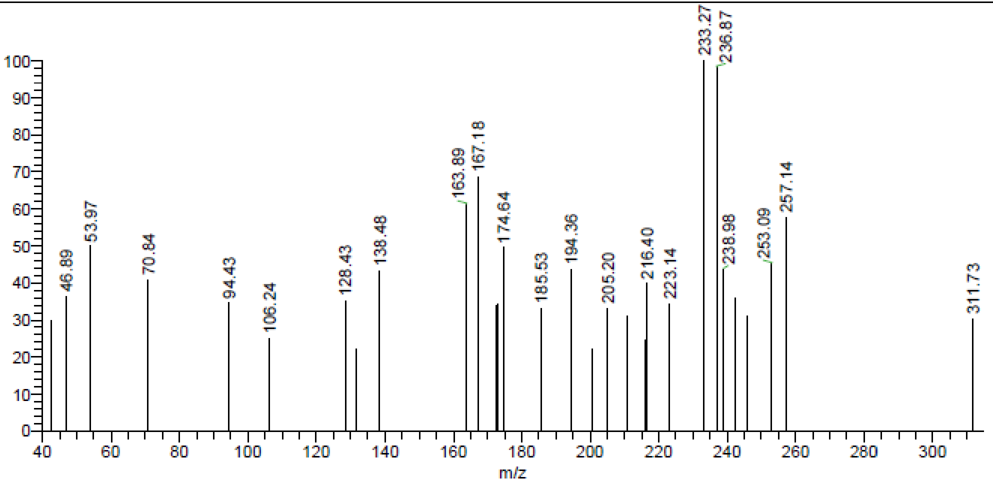

43.20
91.27
77.18
44.30
65.35
53.18
92.22
97.34
105.29
119.33
127.50
67.15
140.35
144.21
167.34
168.35
184.42
207.40
215.89
247.63
251.33
278.35
286.48
305.88
312.54
357.66
371.34
387.54
m/z

40.72
41.76
58.27
60.49
61.89
77.90
82.19
91.40
92.82
100.87
101.76
113.09
118.44
131.00
146.40
152.28
166.08
170.28
184.27
224.36
246.04
260.36
274.81
278.35
322.71
329.88
334.89
336.62
350.13
352.59
m/z

46.89
53.97
70.84
94.43
106.24
128.43
138.48
163.89
167.18
174.64
185.53
194.36
205.20
216.40
223.14
233.27
236.87
238.98
253.09
257.14
311.73
m/z

10e

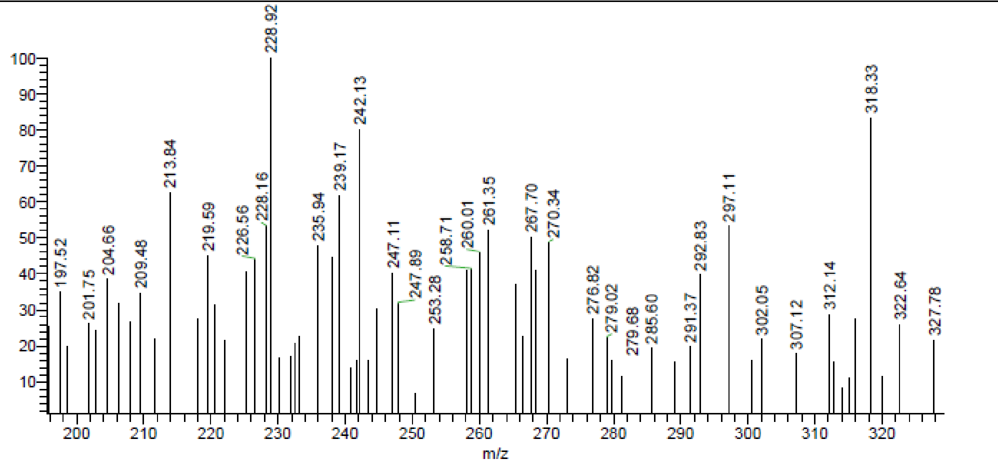
197.52
201.75
204.66
209.48
213.84
219.59
226.56
228.16
228.92
235.94
239.17
242.13
247.11
247.89
253.28
258.71
260.01
261.35
267.70
270.34
276.82
279.02
279.68
285.60
291.37
292.83
297.11
302.05
307.12
312.14
318.33
322.64
327.78
m/z

10f

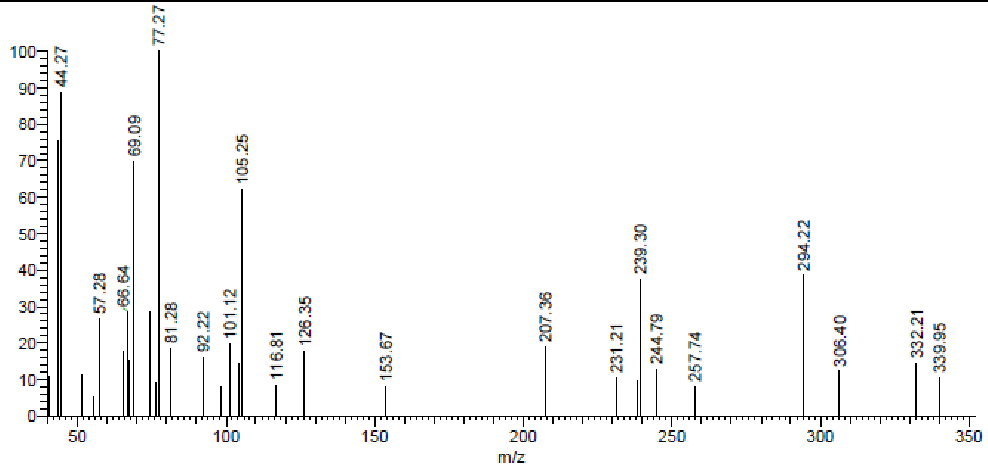
44.27
57.28
66.64
69.09
77.27
81.28
92.22
101.12
105.25
116.81
126.35
153.67
207.36
231.21
239.30
244.79
257.74
294.22
306.40
332.21
339.95
m/z

10g

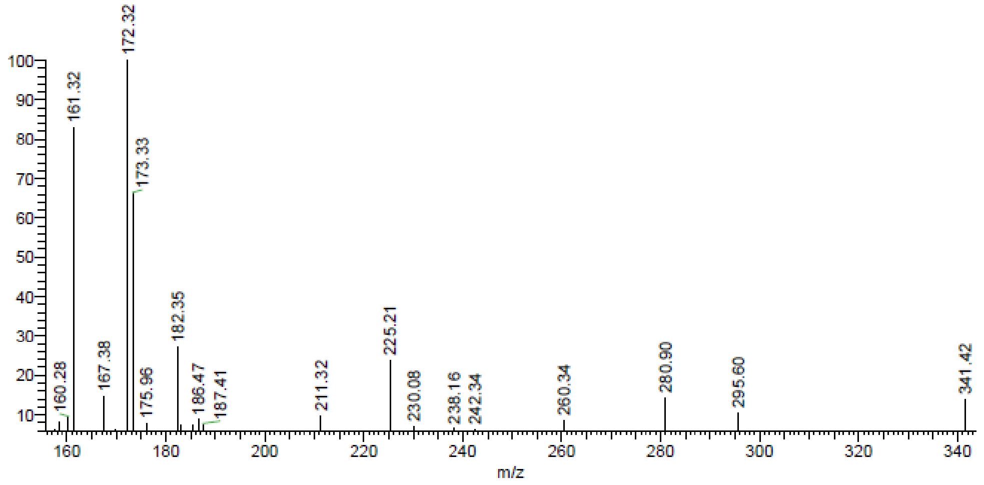
160.28
161.32
167.38
172.32
173.33
175.96
182.35
186.47
187.41
211.32
225.21
230.08
238.16
242.34
260.34
280.90
295.60
341.42
m/z

10h

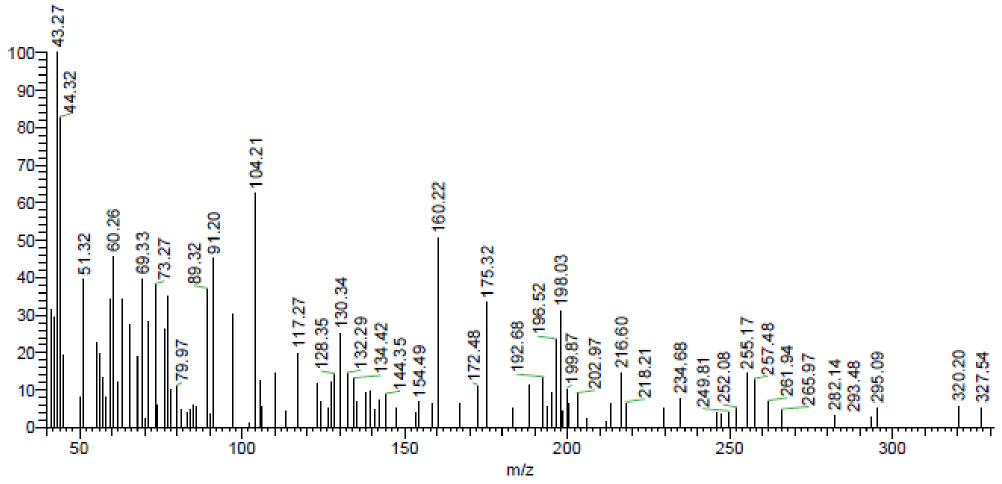
43.27
44.32
51.32
60.26
69.33
73.27
79.97
89.32
91.20
104.21
117.27
128.35
130.34
132.29
134.42
144.35
154.49
160.22
172.48
175.32
192.68
196.52
198.03
199.87
202.97
216.60
218.21
234.68
249.81
252.08
255.17
257.48
261.94
265.97
282.14
293.48
295.09
320.20
327.54
m/z

10i

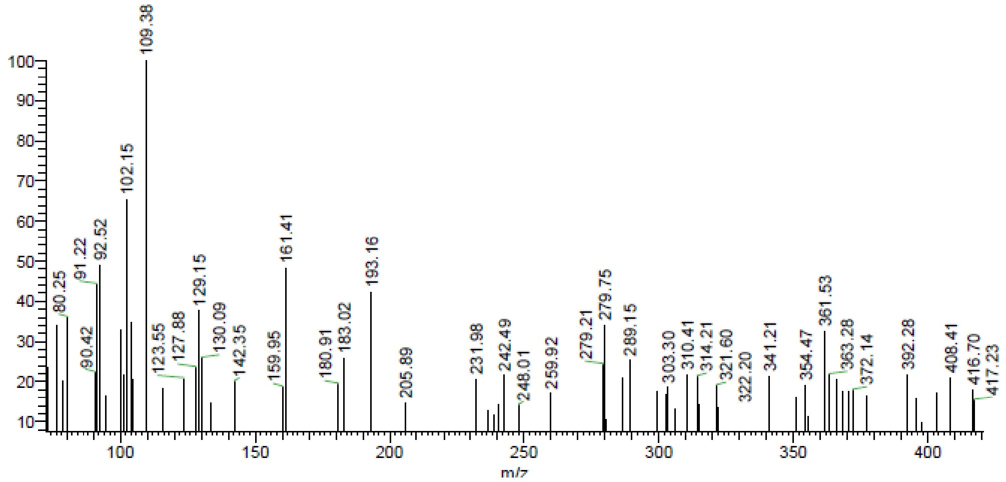
109.38
102.15
92.52
91.22
80.25
90.42
123.55
127.88
129.15
130.09
142.35
159.95
161.41
180.91
183.02
193.16
205.89
231.98
242.49
248.01
259.92
279.21
279.75
289.15
303.30
310.41
314.21
321.60
322.20
341.21
354.47
361.53
363.28
372.14
392.28
408.41
416.70
417.23

10j

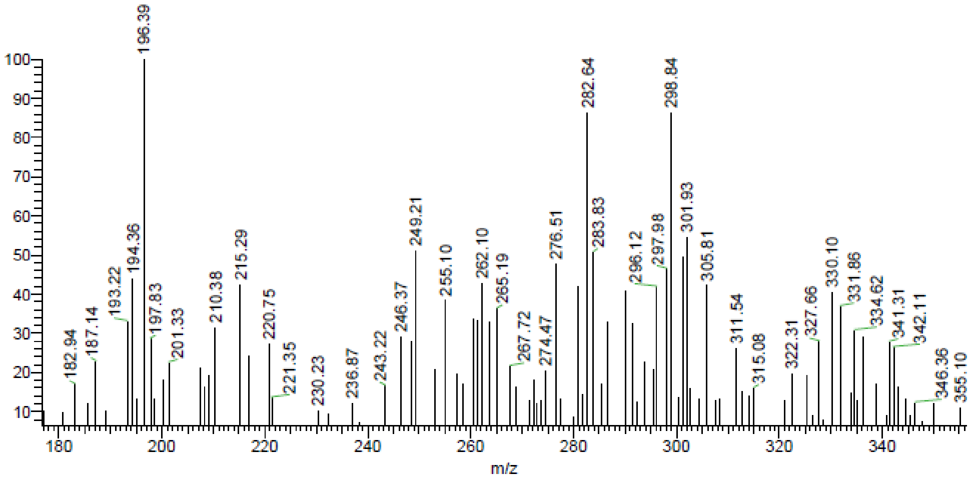
196.39
182.94
187.14
193.22
194.36
197.83
201.33
210.38
215.29
220.75
221.35
230.23
236.87
243.22
246.37
249.21
255.10
262.10
265.19
267.72
274.47
276.51
282.64
283.83
296.12
297.98
298.84
301.93
305.81
311.54
315.08
322.31
327.66
330.10
331.86
334.62
341.31
342.11
346.36
355.10
m/z

**10k**	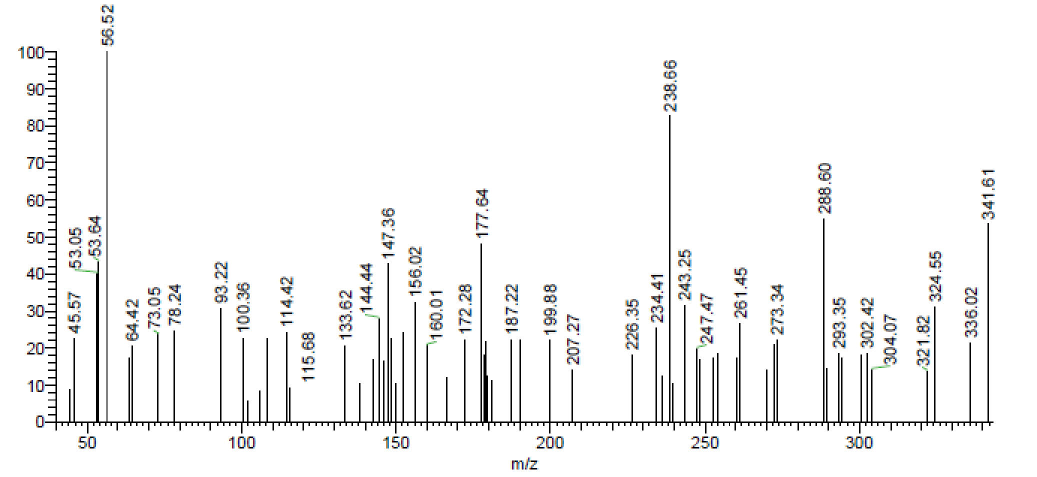
**10l**	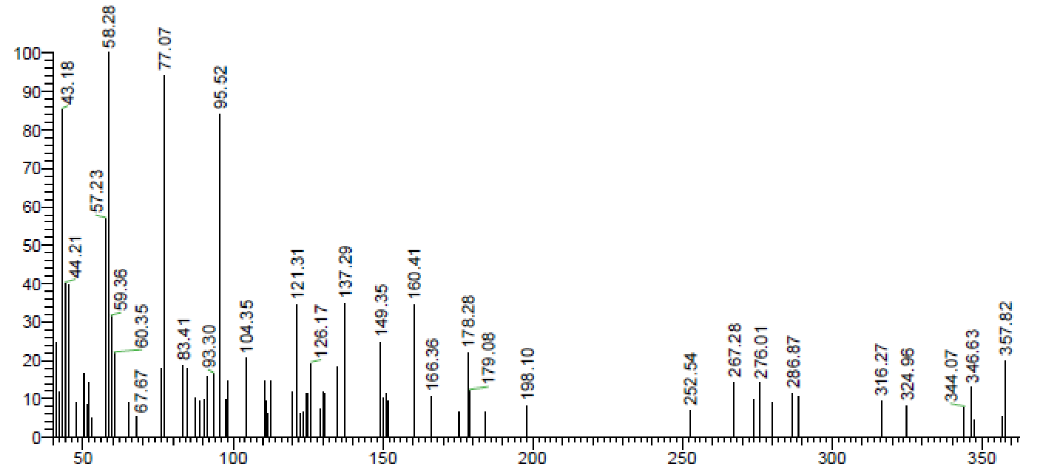

10k: 56.52, 53.05, 53.64, 45.57, 64.42, 73.05, 78.24, 93.22, 100.36, 114.42, 115.68, 133.62, 144.44, 147.36, 156.02, 160.01, 172.28, 177.64, 187.22, 199.88, 207.27, 226.35, 234.41, 238.66, 243.25, 247.47, 261.45, 273.34, 288.60, 293.35, 302.42, 304.07, 321.82, 324.55, 336.02, 341.61
m/z

10l: 58.28, 43.18, 57.23, 44.21, 59.36, 60.35, 67.67, 77.07, 83.41, 93.30, 95.52, 104.35, 121.31, 126.17, 137.29, 149.35, 160.41, 166.36, 178.28, 179.08, 198.10, 252.54, 267.28, 276.01, 286.87, 316.27, 324.96, 344.07, 346.63, 357.82
